# Mediating roles of preterm birth and restricted fetal growth in the relationship between maternal education and infant mortality: A Danish population-based cohort study

**DOI:** 10.1371/journal.pmed.1002831

**Published:** 2019-06-14

**Authors:** Yongfu Yu, Zeyan Liew, Aolin Wang, Onyebuchi A. Arah, Jialiang Li, Jørn Olsen, Sven Cnattingius, Guoyou Qin, Carsten Obel, Bo Fu, Jiong Li

**Affiliations:** 1 Department of Clinical Epidemiology, Aarhus University, Aarhus, Denmark; 2 Department of Epidemiology, Fielding School of Public Health, University of California, Los Angeles, Los Angeles, California, United States of America; 3 Great Ormond Street Institute of Child Health, University College London, London, United Kingdom; 4 Administrative Data Research Centre for England, University College London, London, United Kingdom; 5 Department of Environmental Health Sciences, Yale School of Public Health, New Haven, Connecticut, United States of America; 6 Yale Center for Perinatal, Pediatric, and Environmental Epidemiology, Yale School of Public Health, New Haven, Connecticut, United States of America; 7 Program on Reproductive Health and the Environment, Department of Obstetrics, Gynecology and Reproductive Sciences, University of California, San Francisco, San Francisco, California, United States of America; 8 Institute for Computational Health Sciences, University of California, San Francisco, San Francisco, California, United States of America; 9 Department of Statistics, College of Letters and Science, University of California, Los Angeles, Los Angeles, California, United States of America; 10 Duke-NUS Medical School, National University of Singapore, Singapore; 11 Department of Statistics & Applied Probability, National University of Singapore, Singapore; 12 Clinical Epidemiology Unit, Department of Medicine, Solna, Karolinska University Hospital, Karolinska Institute, Stockholm, Sweden; 13 Department of Biostatistics, School of Public Health, Key Laboratory of Public Health Safety, Fudan University, Shanghai, China; 14 Section for General Medical Practice, Department of Public Health, Aarhus University, Aarhus, Denmark; 15 School of Data Science, Fudan University, Shanghai, China; 16 Ministry of Education–Shanghai Key Laboratory of Children’s Environmental Health, Xinhua Hospital, Shanghai Jiao Tong University School of Medicine, Shanghai, China; London School of Hygiene and Tropical Medicine, UNITED KINGDOM

## Abstract

**Background:**

Socioeconomic disparities in infant mortality have persisted for decades in high-income countries and may have become stronger in some populations. Therefore, new understandings of the mechanisms that underlie socioeconomic differences in infant deaths are essential for creating and implementing health initiatives to reduce these deaths. We aimed to explore whether and the extent to which preterm birth (PTB) and small for gestational age (SGA) at birth mediate the association between maternal education and infant mortality.

**Methods and findings:**

We developed a population-based cohort study to include all 1,994,618 live singletons born in Denmark in 1981–2015. Infants were followed from birth until death, emigration, or the day before the first birthday, whichever came first. Maternal education at childbirth was categorized as low, medium, or high. An inverse probability weighting of marginal structural models was used to estimate the controlled direct effect (CDE) of maternal education on offspring infant mortality, further split into neonatal (0–27 days) and postneonatal (28–364 days) deaths, and portion eliminated (PE) by eliminating mediation by PTB and SGA. The proportion eliminated by eliminating mediation by PTB and SGA was reported if the mortality rate ratios (MRRs) of CDE and PE were in the same direction. The MRRs between maternal education and infant mortality were 1.63 (95% CI 1.48–1.80, *P <* 0.001) and 1.19 (95% CI 1.08–1.31, *P <* 0.001) for low and medium versus high education, respectively. The estimated proportions of these total associations eliminated by reducing PTB and SGA together were 55% (MRR_PE_ = 1.27, 95% CI 1.15–1.40, *P <* 0.001) for low and 60% (MRR_PE_ = 1.11, 95% CI 1.01–1.22, *P =* 0.037) for medium versus high education. The proportions eliminated by eliminating PTB and SGA separately were, respectively, 46% and 11% for low education (versus high education) and 48% and 13% for medium education (versus high education). PTB and SGA together contributed more to the association of maternal educational disparities with neonatal mortality (proportion eliminated: 75%–81%) than with postneonatal mortality (proportion eliminated: 21%–23%). Limitations of the study include the untestable assumption of no unmeasured confounders for the causal mediation analysis, and the limited generalizability of the findings to other countries with varying disparities in access and quality of perinatal healthcare.

**Conclusions:**

PTB and SGA may play substantial roles in the relationship between low maternal education and infant mortality, especially for neonatal mortality. The mediating role of PTB appeared to be much stronger than that of SGA. Public health strategies aimed at reducing neonatal mortality in high-income countries may need to address socially related prenatal risk factors of PTB and impaired fetal growth. The substantial association of maternal education with postneonatal mortality not accounted for by PTB or SGA could reflect unaddressed educational disparities in infant care or other factors.

## Abstract 2

Bo Fu and colleagues analyse factors that may mediate associations between maternal education and child health.

## Author summary

**Why was this study done?:**

- Infant mortality in high-income countries has decreased in recent decades.
- However, socioeconomic inequality in infant mortality remains and may have become stronger in some populations.
- This study provides in-depth knowledge on the underlying pathways from maternal socioeconomic inequality to infant mortality, which is important for developing preventive strategies to reduce potentially preventable deaths.

**What did the researchers do and find?:**

- We conducted a national population-based cohort study that included all 1.99 million live singletons born in Denmark in 1981–2015.
- Using modern modeling methods for causal mediation analysis, we found that preterm birth and restricted fetal growth may mediate the association between low maternal education and infant mortality.
- The mediated effects through preterm birth and restricted fetal growth were substantial for neonatal mortality but not for postneonatal mortality. The effect through preterm birth seemed to be greater than that through restricted fetal growth.

**What do these findings mean?:**

- Public health strategies could aim to reduce educational differences in the death of newborns in high-income countries by addressing socially related risk factors for preterm birth and impaired fetal growth, such as the accessibility and quality of maternity and perinatal care, or maternal lifestyle factors.
- The substantial direct impact of low maternal education on postneonatal death not explained by preterm birth or restricted fetal growth could be due to unaddressed educational inequality in infant care or other factors such as housing conditions, the quality of medical care, or the use of specialized medical care.

## Introduction

Infant mortality in high-income countries has decreased over the last decades [1]. Nevertheless, socioeconomic disparities in infant mortality persist [2–5] and may even have become stronger in some populations [6,7]. A new understanding of the mechanisms that underlie socioeconomic differences in infant deaths is essential to guide health initiatives to reduce potentially preventable deaths.

Preterm birth (PTB), small for gestational age (SGA), and low birth weight (LBW) are not only main risk indicators for infant morbidity and mortality, particularly during the neonatal period, but are also associated with socioeconomic disadvantage [4,8–14]. The primary causes of LBW are PTB and fetal growth restriction [12]. It is possible that the association between socioeconomic disadvantage and infant mortality is mediated through PTB and SGA as a proxy of fetal growth restriction [12,15]. PTB and fetal growth restriction may reflect different mechanisms, and the risk factors for PTB and fetal growth restriction are different [16,17]. The risk of infant mortality is higher among preterm-born infants than SGA infants [12,18]. However, there is a lack of research addressing the possible mediating role of PTB and SGA in the pathway linking maternal socioeconomic disadvantage to an increased risk of infant mortality. Causal mediation analysis may help advance our understanding of when and how socioeconomic disadvantage has a large impact on infant mortality. If the mediating pathway through PTB and SGA plays a major role, policies should focus on the health of women before and during pregnancy to reduce the risk of PTB and SGA. Otherwise, interventions targeting other factors should be explored.

Specifically, using data from Danish registers, we aimed to use causal mediation analysis to examine whether and the extent to which PTB and SGA mediate the association of maternal education with infant mortality. Analyses were stratified by the time (neonatal or postneonatal period) and cause (disease or external cause) of death.

## Methods

### Ethics statement

The study was approved by the Data Protection Agency and Research Ethics Committee of the Central Region in Denmark. By Danish law, no informed consent is required for a register-based study using anonymized data. The prespecified analysis plan is given in S1 Text.

### Study design and participants

The unique personal identification number in Denmark allows accurate linkage of personal data across national registers. The Danish Civil Registration System was linked to the Danish National Patient Register, the Danish Medical Birth Register, the Danish Register of Causes of Death, and the Danish Integrated Database for Labor Market Research [19]. There were 2,096,320 live singleton births registered in Denmark in 1981–2015. We excluded infants with birthweight less than 500 grams or gestational age at birth less than 22 weeks, as criteria for registration of live births and stillbirth vary internationally [20]. We also excluded infants with missing information on maternal education. Follow-up started at birth and ended at death, emigration, or the day before the first birthday, whichever came first.

### Maternal education

The Danish Integrated Database for Labor Market Research [19] provided the information on maternal education, which was measured as the highest level of education attained at childbirth and categorized as low (primary and lower secondary education), medium (upper secondary education or academy profession degree), or high (university education at bachelor’s degree level or higher).

### Outcomes

The outcome of interest was all-cause infant mortality (0–364 days), which was divided into neonatal mortality (0–27 days) and postneonatal mortality (28–364 days). We also investigated mortality by cause of death (death due to disease or medical condition, or external cause), as well as death due to certain conditions originating in the perinatal period [ICD-8 codes 760–779 and ICD-10 P00–P96] and congenital malformations [ICD-8 740–759 and ICD-10 Q00–Q99], according to the European Shortlist for Causes of Death [21].

### Potential mediators

Birth weight and gestational age were extracted from the Danish Medical Birth Register [22]. Gestational age was estimated by the date of last menstrual period and, for all pregnancies since 1995, was adjusted, if necessary, by ultrasonography. The mediators were dichotomized as PTB (1 if gestational age at birth < 37 weeks, 0 otherwise) and SGA (1 if birthweight below the 10th percentile for infants of the same gestational age, sex, and birth year, 0 otherwise) in the main analyses. We also used finer categorizations of PTB (<28, 28–31, 32–36, or 37+ weeks) and SGA (birthweight below the 3rd, between the 3rd and 10th, or above the 10th percentile for infants of the same gestational age, sex, and birth year).

### Covariates

Potential confounders (covariates) included maternal age at birth (<20, 20–24, 25–29, 30–34, ≥35 years), parity (1, 2, 3, ≥4), maternal smoking at delivery (yes, no), maternal cohabitation at delivery (single, cohabitation), maternal residence at delivery (Copenhagen, city with ≥100,000 inhabitants, small town/other), diagnosis of congenital malformation of the infant (yes, no), infant sex (male, female), and birth year of the infant (in 5-year intervals). Maternal smoking and congenital malformation and sex of the child were used to adjust for the confounding between the mediators and the outcome when estimating controlled direct effect (CDE); we preserved their potential mediating roles between the exposure and the outcome (in the statistical analysis).

### Statistical analysis

The approach for causal mediation analysis was based on a counterfactual framework whereby the total effect (TE) can be decomposed into controlled direct effect (CDE) and portion eliminated (PE) [23–25]. The CDE captured the influence of maternal education on infant (neonatal and postneonatal) mortality if the link between maternal education and the mediator (PTB or SGA) was prevented or removed hypothetically. This simulated a scenario wherein the sample distributions of the mediator were no longer dependent on maternal education. PE, the difference between TE and CDE, measured the portion of the TE of maternal education that would be eliminated by eliminating the mediator. TE and CDE were estimated using inverse-probability-weighted marginal structural models (MSMs) [26]. For the MSM for the TE, we used weighted regressions of infant mortality on maternal education. The weight, which is called the inverse-probability-of-treatment weight (IPTW), was estimated for each mother in the sample as the ratio of (i) the estimated marginal probability of the mother’s actual educational attainment to (ii) the estimated probability of each mother’s actual educational attainment conditional on their aforementioned covariates (excluding maternal smoking, offspring sex, congenital malformation of offspring, PTB, and SGA). The IPTW simulates the scenario wherein these covariates, which could be confounders, are no longer associated with maternal education, thus eliminating any confounding by these covariates. To estimate the CDE, the corresponding MSM used a product of the IPTW for maternal education and an additional inverse-probability-of-mediators weight (IPMW). The IPMW was estimated for each infant in the sample as the ratio of (i) the estimated marginal probability of the infant’s actual PTB and SGA to (ii) the estimated probability of each infant’s actual PTB and SGA conditional on their aforementioned covariates. The IPMW simulates the scenario wherein the mediators (PTB and SGA) are no longer associated with maternal education, thus eliminating any mediation by the mediators. The PE was subsequently estimated from the model for TE offsetting the estimated CDE. We considered possible exposure–mediator and mediator–mediator interactions. As the mediator–mediator interactions in causal mediation analysis with multiple mediators were null, the final models included only exposure–mediator interactions. We estimated mortality rate ratios (MRRs) with their 95% confidence intervals (CIs) based on robust variance estimation. The proportion of the TE eliminated through the 2 mediators, i.e., the proportion eliminated ([MRR_TE_−MRR_CDE_]/[MRR_TE_− 1]), was reported if the MRRs of CDE and PE were in the same direction [23,27]. We first assessed the mediating role of PTB and SGA separately, i.e., one mediator at a time. Then we analyzed PTB and SGA together as a joint mediator, i.e., not separating their individual contributions. We also examined the mediating roles of PTB in non-SGA infants as well as the mediating role of SGA in term-born infants. The mediation analyses were performed according to birth year (1981–2015 in 5-year intervals). We used the missing-value indicator method to deal with missing values, such that missing values were treated as a separate category. A detailed description of the inverse probability weighting approach for causal mediation analysis is given in S2 Text. We also performed additional mediation analysis using a traditional approach (“with and without mediator”) [28].

### Sensitivity analysis

We performed a sensitivity analysis to assess and adjust for violations of the uncontrolled confounding assumption [23,29]. Specifically, we considered a binary unmeasured confounding variable *U* indicating a common cause of PTB, SGA, and infant mortality (e.g., maternal alcohol use, maternal body mass index, prenatal care, maternal psychological stress in adolescence, or maternal school attendance [30,31]). We assumed that among infants with normal gestational age and normal birth weight for gestational age, the prevalence of *U* was 20% for the low maternal education group, 30% for the medium maternal education group, and 40% for the high maternal education group. We also considered a simplified assumption that the prevalence of *U* was the same among different maternal education groups. We evaluated the impact of unmeasured mediator–outcome confounding in 2 settings: (i) moderate confounding, where we considered if *U* increased the likelihood of infant mortality by a factor of 1.5, and (ii) strong confounding, where we considered if *U* increased the likelihood of infant mortality by a factor of 2.5 [30]. We also performed analysis with an additional adjustment for maternal country of origin. All analyses were conducted using SAS 9.4 (SAS Institute, Cary, NC, US) and Stata 13 (StataCorp, College Station, TX, US).

## Results

The study included 1,994,618 infants. Excluded infants (*N =* 101,702, 4.85%) had a higher infant mortality rate (1.46%) than included infants (0.43%) and tended to have a younger mother. Overall, infant mortality was 4.3 per 1,000 births (8,563 died), and 61.86% of deaths occurred in the neonatal period. Compared with infants of mothers with medium or high education, infants of mothers with low education were more likely to have increased risk of death, to have PTB, to be SGA, and to be born of mothers of younger childbearing age, with higher parity, who lived alone, and who were from small towns (Table 1).

**Table 1:** Baseline characteristics for mother and child across maternal education at birth.

Characteristic	Maternal education group			Total
	Low	Medium	High	
Number of infants	520,642 (26.1)	893,388 (44.8)	580,588 (29.1)	1,994,618
Infant deaths	3,398 (0.7)	3,452 (0.4)	1,713 (0.3)	8,563 (0.4)
Preterm birth				
<37 weeks	27,855 (5.4)	37,354 (4.2)	19,761 (3.4)	84,970 (4.3)
37+ weeks	492,787 (94.7)	856,034 (95.8)	560,827 (96.6)	1,909,648 (95.7)
Small for gestational age				
Yes	64,390 (12.4)	82,894 (9.3)	44,961 (7.7)	192,245 (9.6)
No	456,252 (87.6)	810,494 (90.7)	535,627 (92.3)	1,802,373 (90.4)
Sex				
Boy	267,352 (51.4)	458,152 (51.3)	298,551 (51.4)	1,024,055 (51.3)
Girl	253,290 (48.7)	435,236 (48.7)	282,037 (48.6)	970,563 (48.7)
Congenital malformation				
No	508,165 (97.6)	873,171 (97.7)	567,462 (97.7)	1,948,798 (97.7)
Yes	12,477 (2.4)	20,217 (2.3)	13,126 (2.3)	45,820 (2.3)
Maternal age at birth (years)				
<20	38,843 (7.5)	3,240 (0.4)	93 (0.0)	42,176 (2.1)
20–24	169,911 (32.6)	153,684 (17.2)	10,757 (1.9)	334,352 (16.8)
25–29	169,311 (32.5)	370,095 (41.4)	191,484 (33.0)	730,890 (36.6)
30–34	96,893 (18.6)	260,190 (29.1)	254,203 (43.8)	611,286 (30.7)
35+	45,684 (8.8)	106,179 (11.9)	124,051 (21.4)	275,914 (13.8)
Parity				
1	220,198 (42.3)	419,604 (47.0)	252,475 (43.5)	892,277 (44.7)
2	178,464 (34.3)	334,980 (37.5)	230,079 (39.6)	743,523 (37.3)
3	81,080 (15.6)	107,746 (12.1)	81,029 (14.0)	269,855 (13.5)
≥4	40,900 (7.9)	31,058 (3.5)	17,005 (2.9)	88,963 (4.5)
Maternal residence at birth				
Copenhagen	47,813 (9.2)	80,670 (9.0)	93,635 (16.1)	222,118 (11.1)
City with ≥100,000 inhabitants	58,696 (11.3)	106,049 (11.9)	94,289 (16.2)	259,034 (13.0)
Small town/other	414,133 (79.5)	706,669 (79.1)	392,664 (67.6)	1,513,466 (75.9)
Maternal cohabitation at birth				
Single	291,133 (55.9)	408,679 (45.7)	229,668 (39.6)	929,480 (46.6)
Cohabitation	229,509 (44.1)	484,709 (54.3)	350,920 (60.4)	1,065,138 (53.4)
Maternal smoking at birth^a^				
No	175,642 (56.4)	529,843 (77.3)	426,139 (89.6)	1,131,624 (76.9)
Yes	122,621 (39.4)	131,263 (19.1)	36,115 (7.6)	289,999 (19.7)
Unknown	13,054 (4.2)	24,655 (3.6)	13,165 (2.8)	50,874 (3.5)
Birth year				
1981–1985	106,293 (20.4)	87,920 (9.8)	47,592 (8.2)	241,805 (12.1)
1986–1990	103,032 (19.8)	119,707 (13.4)	57,577 (9.9)	280,316 (14.1)
1991–1995	93,659 (18.0)	157,209 (17.6)	68,389 (11.8)	319,257 (16.0)
1996–2000	72,893 (14.0)	156,065 (17.5)	77,183 (13.3)	306,141 (15.4)
2001–2005	58,160 (11.2)	145,453 (16.3)	97,506 (16.8)	301,119 (15.1)
2006–2010	49,143 (9.4)	127,187 (14.2)	117,568 (20.3)	293,898 (14.7)
2011–2015	37,462 (7.2)	99,847 (11.2)	114,773 (19.8)	252,082 (12.6)

Data are expressed as frequency (percentage). Percentages have been rounded and may not total 100.

^a^Maternal smoking was available from 1991 to 2015.

Regarding the total association of maternal education with mortality, we observed that MRRs decreased with increasing education level (Table 2; S1 Fig). The MRR_TE_ of association with low maternal education (versus high) was 1.63 (95% CI 1.48–1.80, *P <* 0.001) for infant mortality (neonatal and postneonatal), 1.57 (95% CI 1.38–1.78, *P <* 0.001) for neonatal mortality, and 1.75 (95% CI 1.49–2.04, *P <* 0.001) for postneonatal mortality. We found that PTB and SGA were associated with both maternal education and infant mortality, and the association between PTB and infant mortality was much stronger than the association between SGA and infant mortality (S1 Table). Analyses stratified by birth year found a stronger association between low maternal education and infant mortality in recent years (S2 Table).

**Table 2:** The contribution of PTB and SGA in explaining the association between maternal education and infant mortality.

Mediator	Period	Education	Number of deaths	Rate/10^2^ pys	MRR_TE_	*P* value	MRR_CDE_	*P* value	MRR_PE_	*P* value	Proportion eliminated
PTB	Infant (<1 year)	Low	3,398	6.58	1.63 (1.48–1.80)	0.000	1.34 (1.21–1.49)	0.000	1.22 (1.10–1.34)	0.000	46%
		Medium	3,452	3.89	1.19 (1.08–1.31)	0.000	1.10 (0.99–1.22)	0.070	1.08 (0.99–1.19)	0.094	48%
		High	1,713	2.97	1.00 (reference)						
	Neonatal (0–27 days)	Low	1,944	50.67	1.57 (1.38–1.78)	0.000	1.20 (1.05–1.37)	0.007	1.30 (1.15–1.48)	0.000	64%
		Medium	2,268	34.42	1.18 (1.05–1.33)	0.006	1.07 (0.94–1.21)	0.330	1.11 (0.99–1.25)	0.087	64%
		High	1,158	27.03	1.00 (reference)						
	Postneonatal (28–364 days)	Low	1,454	3.04	1.75 (1.49–2.04)	0.000	1.62 (1.36–1.92)	0.000	1.08 (0.93–1.26)	0.328	17%
		Medium	1,184	1.44	1.21 (1.04–1.41)	0.015	1.17 (0.98–1.38)	0.075	1.04 (0.89–1.21)	0.650	20%
		High	555	1.04	1.00 (reference)						
SGA	Infant (<1 year)	Low	3,398	6.58	1.63 (1.48–1.80)	0.000	1.56 (1.42–1.72)	0.000	1.05 (0.95–1.15)	0.377	11%
		Medium	3,452	3.89	1.19 (1.08–1.31)	0.000	1.17 (1.06–1.28)	0.001	1.02 (0.93–1.12)	0.662	13%
		High	1,713	2.97	1.00 (reference)						
	Neonatal (0–27 days)	Low	1,944	50.67	1.57 (1.38–1.78)	0.000	1.50 (1.32–1.70)	0.000	1.05 (0.92–1.19)	0.478	12%
		Medium	2,268	34.42	1.18 (1.05–1.33)	0.006	1.15 (1.02–1.29)	0.018	1.03 (0.91–1.16)	0.661	17%
		High	1,158	27.03	1.00 (reference)						
	Postneonatal (28–364 days)	Low	1,454	3.04	1.75 (1.49–2.04)	0.000	1.68 (1.44–1.96)	0.000	1.04 (0.89–1.21)	0.648	8%
		Medium	1,184	1.44	1.21 (1.04–1.41)	0.015	1.20 (1.03–1.39)	0.020	1.01 (0.87–1.18)	0.902	6%
		High	555	1.04	1.00 (reference)						
PTB and SGA	Infant (<1 year)	Low	3,398	6.58	1.63 (1.48–1.80)	0.000	1.28 (1.16–1.42)	0.000	1.27 (1.15–1.40)	0.000	55%
		Medium	3,452	3.89	1.19 (1.08–1.31)	0.000	1.08 (0.97–1.19)	0.141	1.11 (1.01–1.22)	0.037	60%
		High	1,713	2.97	1.00 (reference)						
	Neonatal (0–27 days)	Low	1,944	50.67	1.57 (1.38–1.78)	0.000	1.14 (1.00–1.30)	0.053	1.37 (1.21–1.56)	0.000	75%
		Medium	2,268	34.42	1.18 (1.05–1.33)	0.006	1.03 (0.91–1.17)	0.553	1.14 (1.01–1.28)	0.032	81%
		High	1,158	27.03	1.00 (reference)						
	Postneonatal (28–364 days)	Low	1,454	3.04	1.75 (1.49–2.04)	0.000	1.58 (1.33–1.87)	0.000	1.11 (0.95–1.29)	0.172	23%
		Medium	1,184	1.44	1.21 (1.04–1.41)	0.015	1.17 (0.99–1.38)	0.076	1.04 (0.89–1.21)	0.614	21%
		High	555	1.04	1.00 (reference)						

Proportion eliminated = (MRR_TE_ − MRR_CDE_)/(MRR_TE_ − 1); proportion eliminated is only presented if the MRRs of CDE and PE were in the same direction.

CDE, controlled direct effect; MRR, mortality rate ratio; PE, portion eliminated; PTB, preterm birth; pys, person-years; SGA, small for gestational age; TE, total effect.

Mediation analysis including PTB and SGA together showed that PTB and SGA played an important role in explaining the link between maternal education and all-cause infant mortality (Table 2; S1 Fig). Compared with high maternal education, the estimated proportion of the total association of education with infant death that could be reduced by “eliminating” the mediating role of PTB and SGA was 55% (MRR_PE_ = 1.27 [95% CI 1.15–1.40, *P <* 0.001]) for low education and 60% (MRR_PE_ = 1.11 [95% CI 1.01–1.22, *P =* 0.037]) for medium education. Regarding neonatal mortality, excess deaths were mainly due to the pathway involving PTB and SGA (low versus high education: proportion eliminated = 75%, MRR_PE_ = 1.37 [95% CI 1.21–1.56, *P <* 0.001]; medium versus high education: proportion eliminated = 81%, MRR_PE_ = 1.14 [95% CI 1.01–1.28, *P =* 0.032]). During the postneonatal period, other pathways, rather than these mediators, may play a major role in explaining the increased rate of deaths (low versus high education: proportion eliminated = 23%, MRR_PE_ = 1.11 [95% CI 0.95–1.29, *P =* 0.172]; medium versus high education: proportion eliminated = 21%, MRR_PE_ = 1.04 [95% CI 0.89–1.21, *P =* 0.614]). Regarding the mediation analyses including one mediator at a time (Table 2; S1 Fig), the mediating role of PTB (low versus high education: proportion eliminated = 46%, MRR_PE_ = 1.22 [95% CI 1.10–1.34, *P <* 0.001]; medium versus high education: proportion eliminated = 48%, MRR_PE_ = 1.08 [95% CI 0.99–1.19, *P =* 0.094]) in the association between maternal education and infant mortality was much stronger than that of SGA (low versus high education: proportion eliminated = 11%, MRR_PE_ = 1.05 [95% CI 0.95–1.15, *P =* 0.377]; medium versus high education: proportion eliminated = 13%, MRR_PE_ = 1.02 [95% CI 0.93–1.12, *P =* 0.662]). Similar patterns were also found for neonatal mortality and postneonatal mortality analyzed separately. A finer categorization of the mediators yielded a stronger mediating impact by PTB and SGA (Table 3).

**Table 3:** The contribution of PTB and SGA in explaining the association between maternal education and infant mortality using a finer categorization of PTB and SGA.

Mediator	Period	Education	MRR_TE_	*P* value	MRR_CDE_	*P* value	MRR_PE_	*P* value	Proportion eliminated
PTB	Infant (<1 year)	Low	1.63 (1.48–1.80)	0.000	1.21 (1.09–1.36)	0.001	1.34 (1.22–1.48)	0.000	66%
		Medium	1.19 (1.08–1.31)	0.000	1.05 (0.95–1.17)	0.338	1.13 (1.03–1.24)	0.011	72%
		High	1.00 (reference)						
	Neonatal (0–27 days)	Low	1.57 (1.38–1.78)	0.000	1.06 (0.92–1.21)	0.441	1.48 (1.31–1.68)	0.000	90%
		Medium	1.18 (1.05–1.33)	0.006	1.02 (0.89–1.16)	0.800	1.16 (1.03–1.31)	0.013	90%
		High	1.00 (reference)						
	Postneonatal (28–364 days)	Low	1.75 (1.49–2.04)	0.000	1.55 (1.30–1.85)	0.000	1.12 (0.96–1.31)	0.137	26%
		Medium	1.21 (1.04–1.41)	0.015	1.14 (0.95–1.36)	0.148	1.06 (0.91–1.24)	0.446	33%
		High	1.00 (reference)						
SGA	Infant (<1 year)	Low	1.63 (1.48–1.80)	0.000	1.55 (1.40–1.70)	0.000	1.06 (0.96–1.16)	0.282	14%
		Medium	1.19 (1.08–1.31)	0.000	1.16 (1.06–1.27)	0.002	1.03 (0.94–1.13)	0.569	17%
		High	1.00 (reference)						
	Neonatal (0–27 days)	Low	1.57 (1.38–1.78)	0.000	1.48 (1.30–1.67)	0.000	1.06 (0.93–1.20)	0.361	16%
		Medium	1.18 (1.05–1.33)	0.006	1.14 (1.02–1.28)	0.026	1.04 (0.92–1.17)	0.558	23%
		High	1.00 (reference)						
	Postneonatal (28–364 days)	Low	1.75 (1.49–2.04)	0.000	1.68 (1.44–1.96)	0.000	1.04 (0.89–1.21)	0.617	9%
		Medium	1.21 (1.04–1.41)	0.015	1.20 (1.03–1.39)	0.021	1.01 (0.87–1.18)	0.882	7%
		High	1.00 (reference)						
PTB and SGA	Infant (<1 year)	Low	1.63 (1.48–1.80)	0.000	1.16 (1.04–1.30)	0.007	1.40 (1.27–1.55)	0.000	74%
		Medium	1.19 (1.08–1.31)	0.000	1.03 (0.93–1.15)	0.527	1.15 (1.05–1.26)	0.003	82%
		High	1.00 (reference)						
	Neonatal (0–27 days)	Low	1.57 (1.38–1.78)	0.000	1.00 (0.88–1.15)	0.975	1.56 (1.38–1.77)	0.000	100%
		Medium	1.18 (1.05–1.33)	0.006	0.99 (0.87–1.13)	0.877	1.19 (1.06–1.34)	0.003	100%
		High	1.00 (reference)						
	Postneonatal (28–364 days)	Low	1.75 (1.49–2.04)	0.000	1.54 (1.30–1.83)	0.000	1.13 (0.97–1.32)	0.114	28%
		Medium	1.21 (1.04–1.41)	0.015	1.15 (0.97–1.37)	0.100	1.05 (0.90–1.22)	0.545	27%
		High	1.00 (reference)						

Proportion eliminated = (MRR_TE_ − MRR_CDE_)/(MRR_TE_ − 1); proportion eliminated is only presented if the MRRs of CDE and PE were in the same direction. A finer categorization of PTB (<28, 28–31, 32–36, 37+ weeks) and SGA (birthweight below the 3rd, between the 3rd and 10th, or above the 10th percentile for infants of the same gestational age, sex, and birth year).

CDE, controlled direct effect; MRR, mortality rate ratio; PE, portion eliminated; PTB, preterm birth; SGA, small for gestational age; TE, total effect.

Mediation analyses stratified by birth year yielded results similar to those from the main analyses, although the estimated proportion of the total association between maternal education and infant death reduced by eliminating the mediating roles of PTB and SGA seemed to decrease in recent years (S2 and S3 Tables). Analyses restricted to non-SGA infants on the mediating role of PTB found a similar pattern as the analyses considering PTB and SGA together (S4 Table). We found that SGA weakly mediated the association between maternal education and infant mortality among term-born infants (S5 Table).

Deaths due to diseases accounted for 96.8% (8,292 died) of all infant deaths, and the pattern observed for infant deaths due to diseases was similar to that of all-cause mortality (Table 4). Similar patterns were also found for deaths due to certain conditions originating in the perinatal period and congenital malformations (S6 and S7 Tables). Especially for infant deaths due to certain conditions originating in the perinatal period, a substantial mediating impact through PTB and SGA together was found (low versus high education: proportion mediated = 76%, MRR_PE_ = 1.49 [95% CI 1.26–1.76, *P <* 0.001]). A much smaller portion of deaths due to external causes was found to be mediated by PTB and SGA (S8 Table).

**Table 4:** The combined contribution of PTB and SGA in explaining the association between maternal education and infant mortality due to diseases.

Mediator	Period	Education	Number of deaths	Rate/10^2^ pys	MRR_TE_	*P* value	MRR_CDE_	*P* value	MRR_PE_	*P* value	Proportion eliminated
PTB	Infant (<1 year)	Low	3,288	6.37	1.62 (1.47–1.79)	0.000	1.33 (1.19–1.48)	0.000	1.22 (1.11–1.35)	0.000	47%
		Medium	3,340	3.76	1.18 (1.07–1.30)	0.001	1.09 (0.98–1.21)	0.108	1.08 (0.99–1.19)	0.095	51%
		High	1,664	2.88	1.00 (reference)						
	Neonatal (0–27 days)	Low	1,924	50.15	1.57 (1.38–1.78)	0.000	1.20 (1.05–1.37)	0.009	1.31 (1.15–1.48)	0.000	65%
		Medium	2,231	33.86	1.17 (1.04–1.32)	0.009	1.06 (0.93–1.20)	0.392	1.11 (0.98–1.25)	0.091	66%
		High	1,145	26.72	1.00 (reference)						
	Postneonatal (28–364 days)	Low	1,364	2.86	1.73 (1.48–2.03)	0.000	1.60 (1.34–1.91)	0.000	1.08 (0.92–1.27)	0.326	18%
		Medium	1,109	1.35	1.20 (1.02–1.41)	0.024	1.16 (0.97–1.38)	0.109	1.04 (0.89–1.22)	0.643	22%
		High	519	0.97	1.00 (reference)						
SGA	Infant (<1 year)	Low	3,288	6.37	1.62 (1.47–1.79)	0.000	1.55 (1.41–1.71)	0.000	1.05 (0.95–1.16)	0.375	12%
		Medium	3,340	3.76	1.18 (1.07–1.30)	0.001	1.16 (1.05–1.27)	0.002	1.02 (0.93–1.12)	0.655	14%
		High	1,664	2.88	1.00 (reference)						
	Neonatal (0–27 days)	Low	1,924	50.15	1.57 (1.38–1.78)	0.000	1.50 (1.32–1.70)	0.000	1.05 (0.92–1.19)	0.477	12%
		Medium	2,231	33.86	1.17 (1.04–1.32)	0.009	1.14 (1.01–1.28)	0.028	1.03 (0.91–1.16)	0.654	18%
		High	1,145	26.72	1.00 (reference)						
	Postneonatal (28–364 days)	Low	1,364	2.86	1.73 (1.48–2.03)	0.000	1.67 (1.42–1.96)	0.000	1.04 (0.88–1.22)	0.645	9%
		Medium	1,109	1.35	1.20 (1.02–1.41)	0.024	1.19 (1.02–1.39)	0.031	1.01 (0.86–1.18)	0.904	6%
		High	519	0.97	1.00 (reference)						
PTB and SGA	Infant (<1 year)	Low	3,288	6.37	1.62 (1.47–1.79)	0.000	1.26 (1.14–1.40)	0.000	1.28 (1.16–1.42)	0.000	58%
		Medium	3,340	3.76	1.18 (1.07–1.30)	0.001	1.07 (0.96–1.18)	0.217	1.11 (1.01–1.22)	0.035	63%
		High	1,664	2.88	1.00 (reference)						
	Neonatal (0–27 days)	Low	1,924	50.15	1.57 (1.38–1.78)	0.000	1.13 (0.99–1.29)	0.063	1.38 (1.22–1.57)	0.000	76%
		Medium	2,231	33.86	1.17 (1.04–1.32)	0.009	1.03 (0.91–1.17)	0.662	1.14 (1.01–1.28)	0.031	84%
		High	1,145	26.72	1.00 (reference)						
	Postneonatal (28–364 days)	Low	1,364	2.86	1.73 (1.48–2.03)	0.000	1.55 (1.30–1.84)	0.000	1.12 (0.95–1.31)	0.169	25%
		Medium	1,109	1.35	1.20 (1.02–1.41)	0.024	1.15 (0.97–1.37)	0.110	1.04 (0.89–1.22)	0.610	24%
		High	519	0.97	1.00 (reference)						

Deaths due to diseases: ICD-8 codes 000–799 and ICD-10 codes A00–R99. Proportion eliminated = (MRR_TE_ − MRR_CDE_)/(MRR_TE_ − 1); proportion eliminated is only presented if the MRRs of CDE and PE were in the same direction.

CDE, controlled direct effect; MRR, mortality rate ratio; PE, portion eliminated; PTB, preterm birth; pys, person-years; SGA, small for gestational age; TE, total effect.

In addition, we performed sensitivity analyses to assess the potential impact of unmeasured confounding. Under the simplified assumption that the prevalence of *U* was the same in each maternal education group, the CDE was unaffected. However, if the prevalence of *U* was assumed to differ between maternal education groups, the adjusted CDE was higher than the original CDE. Even if the unmeasured confounder was strong enough to increase the likelihood of infant death by 2.5-fold, we still observed the mediating impact of PTB on neonatal mortality (S9 Table). An additional adjustment for maternal country of origin (S10 Table) did not essentially change the results. The results from the traditional approach with and without mediators for mediation analysis differed from those of our MSM approach (S11 Table).

## Discussion

We found that low maternal education was associated with a higher risk of infant mortality in Denmark, and this association was partly mediated by PTB and SGA. The mediatory role of PTB and SGA was substantial in neonatal mortality, while a large direct impact of low maternal education on postneonatal mortality was observed. The mediating effects through PTB were greater than those through SGA.

A number of studies [3,5,9,10,32] have investigated the association between maternal socioeconomic disadvantage and infant mortality, but few of them have attempted to distinguish the relative roles of LBW, PTB, and SGA in the association. A British study [15], using a traditional method to explore the association between social class and infant mortality (“with and without mediator”), reported that LBW was a strong risk factor in the neonatal period but did not seem to play an important role in the postneonatal period. The traditional regression approach, including a potential mediator as a covariate, is easily implemented and understood. However, the traditional mediation analysis method is prone to yielding a flawed conclusion due to exposure–mediator interaction, mediator–outcome confounding, and mediator–outcome confounding affected by the exposure (intermediate confounding) [23,28]. Especially in the context of longitudinal design and time-varying confounders, intermediate confounders may not be rare. In a traditional mediation approach, adjustment for the mediator might lead to a spurious association between the intermediate confounder and the exposure, where the intermediate confounder becomes the confounder between the exposure and the outcome. Adjustment for intermediate confounders is required to prevent such bias, known as collider-stratification bias [23]. However, adjustment for intermediate confounders in a traditional mediation model could block part of the effect of exposure on outcome through the intermediate confounder, resulting in underestimation of the direct effect not through the mediator and overestimation of the mediating effect through the mediator [23,33]. Our findings also suggest that the traditional approach for mediation analysis is probably subject to bias (S11 Table). We used a counterfactual approach for causal mediation analysis that allows decomposing the TE into the CDE and the PE in the presence of exposure–mediator interaction [23]. Inverse-probability-weighted MSMs could address intermediate confounders and estimate the CDE, without blocking the pathway from the exposure to the outcome acting through the intermediate confounders (see S2 Text for details) [23,26]. Moreover, sensitivity analysis techniques based on a causal inference framework can be applied to evaluate the impact of unmeasured confounders [29].

Low socioeconomic status has been associated with PTB, SGA, and LBW [12–14,34,35]. Socioeconomic inequality may lead to differences in prenatal risk factors for PTB and SGA, including the accessibility/quality of maternity/perinatal care, maternal health behaviors, occupational situation, nutrition, and health outcomes [2,35–37]. These prenatal risk factors may have an influence on risks of PTB and fetal growth restriction, thereby leading to an increased risk of infant death [36]. Certain conditions originating in the perinatal period and congenital malformations are the 2 leading causes of infant death [38]. In mediation analyses of infant mortality due to certain conditions originating in the perinatal period and congenital malformations, we found similar mediating patterns as in the mediation analyses for overall infant mortality. PTB, SGA, and LBW are important indirect causes of neonatal deaths [12,36,39], and our results also showed that the mediating roles of PTB and SGA accounted for a large number of excess neonatal deaths related to low maternal education.

In accordance with previous studies, we observed that the total association of maternal education and infant mortality tended to be stronger in the postneonatal period than in the neonatal period [5,32]. Our finding that both the CDE and PE of maternal education on infant mortality differed between the neonatal and the postneonatal period may reflect separate causal pathways. The substantial impact of PTB and SGA on the association of maternal education with neonatal mortality due to diseases may reflect that the excess neonatal deaths are more influenced by prenatal and perinatal mechanisms, like PTB, placental impairment, and impaired fetal growth. Although neonatal mortality has been linked to the quality of obstetric and neonatal care [31], it is less likely that maternal disadvantage would significantly affect the care provided by the neonatal intensive care center in Denmark, where there is universal tax-paid health coverage and the utilization of these services is very high. Disorders related to short gestation and impaired fetal growth are important causes of neonatal death [8], and might partly explain why maternal education mainly acted through the mediating role of PTB and SGA.

In the postneonatal period, substantial direct impact of maternal education on offspring mortality was seen. Although Denmark has universal health coverage for all essential health services, education could reflect differences in both economic and non-economic factors. Higher levels of education are related to active acquisition of health-related knowledge and increased use of special health services. Education also indicates the ability to solve problems and the capacity to deal with stressors [40]. Infants with well-educated mothers are more likely to benefit from optimized use of health and social welfare services and to receive a higher quality of clinical care [31]. A previous study showed that children born to mothers with lower education used fewer general practitioner services and specialist services, and there were considerable differences in the use of telephone consultations with doctors according to maternal education [41]. Therefore, socioeconomic postnatal differences in the environmental and social circumstances of infants are likely to be a vital determinant of postneonatal mortality [5]. It is important to further evaluate this finding in other countries with larger gaps in the quality of and access to postnatal care.

From the public health perspective, the findings from this study improve our understanding of the underlying pathways from maternal education to infant mortality, which should be taken into consideration when designing preventative strategies. The overall educational disparities in infant mortality could possibly be reduced by tackling different intermediates along the pathways. First of all, it is critical to understand and identify socially related prenatal risk factors of PTB and restricted fetal growth. Poor maternal health has been suggested to be strongly associated with adverse birth characteristics [42]. Intervention strategies focused on improving the health of socially and economically disadvantaged women before and during pregnancy to reduce the risk of PTB and SGA may help to prevent neonatal mortality [31,42]. For postneonatal mortality, we found that pathways other than PTB and SGA may play a more critical role. Educational attainment of women is crucial for their access to labor market, income, social resources, financial status, and health behaviors [43]. Education level may have a direct impact on infant health through housing conditions, occupational status, lifestyle, diet, psychosocial stress, compliance with medical advice, the quality of medical care, and the use of specialized medical care [44]. Therefore, efforts to reduce postneonatal deaths need to minimize the link between maternal education and such factors [45]. Reducing these disparities in infant care will require a significant and coordinated effort from different sectors, such as health, housing, labor, and education. Even in a welfare society such as Denmark, pregnant women with low education need more attention and resources to address socially related risk factors to improve infant health in general, and reduce infant mortality.

### Strengths and limitations

The unique methodological strength of this study is that we have sufficient and good quality data for performing modern causal mediation analysis in a large study population. Causal mediation analysis can help to explain the mechanisms behind the impact of maternal socioeconomic disadvantage on infant mortality and inform the process of designing public health interventions that will prevent infant deaths. Our study estimated the joint mediating effect of PTB and SGA using the weighting-based approach for causal mediation analysis, which does not necessarily require knowing the ordering of the mediators and is able to address the possibility that the mediators affect each other. It allowed for possible mediator–exposure and mediator–mediator interactions. Furthermore, our large population-based cohort study with almost complete follow-up provided high-quality prospective data and minimized the potential influence of selection bias and recall bias.

The results of the study should be carefully interpreted given the following limitations. First, for valid inferences, causal mediation analysis requires the untestable assumption of there being no unmeasured confounders of the association between infant mortality, maternal education, and the mediators [23,28,33]. Although we adjusted for a wide range of confounders, we cannot exclude residual confounding by unmeasured maternal demographic and lifestyle factors, such as alcohol consumption, body mass index, physical inactivity, psychological stress in adolescence, or school attendance. We applied sensitivity analysis [23,29] to assess the potential impact of unmeasured mediator–outcome confounders on the main results. The results from sensitivity analysis indicated that fairly substantial confounding would be required to explain away our reported results. In addition, some of these unmeasured variables can also be considered as mediators in the pathway between maternal education and infant mortality, in which case they should not be included in the model. Second, ethnicity could be a confounder for both exposure–outcome association and exposure–mediator association. In the US and several European countries, ethnic minority groups have consistently lower birthweight than the predominant ethnic group. Ethnic disparity is likely due to variations in access to antenatal care and socio-environmental factors, such as workload, stress, and diet [46–48]. However, ethnic disparity is unlikely to be a major concern in Denmark as 91.8% of Danish women are of Danish descent [49], and analyses with additional control for country of origin of the mother (>90% of mothers originally from Denmark [50]) did not change the results. Third, average maternal education levels have increased between 1981 and 2015 in Denmark. The women with only primary and lower secondary education may have become a highly selected group at the end of the period due to increased average education level. Therefore, the stronger association between maternal education and infant mortality observed in recent years could be due to a higher prevalence of risk factors for infant mortality among women with low education. In addition, the same educational attainment does not necessarily reflect the same classifying functions in different calendar periods. The improved educational attainment over the past decades may influence the associations observed in the study. However, mediation analyses stratified by birth year interval found that the results in different intervals were similar to those from the main analyses using data during the whole study period. Finally, our study is based on register data from Denmark, which has universal health coverage for all essential healthcare services. It is important to evaluate these findings in countries with larger disparities in terms of access and the quality of perinatal healthcare.

### Conclusions

The association between low maternal education and infant mortality mediated through PTB and SGA was large for neonatal mortality but small for postneonatal mortality. Public health preventive strategies for education-related neonatal mortality in high-income countries may need to address the socially related prenatal risk factors of PTB and impaired fetal growth. On the other hand, the substantial direct association of maternal education with deaths not accounted for by PTB and SGA during the postneonatal period could reflect unaddressed educational disparities in infant care or other factors.

## Supporting information

S1 Checklist STROBE checklist for reporting cohort studies. (DOCX)

S1 Fig The contribution of preterm birth and small for gestational age in explaining the association between maternal education and infant mortality in Denmark. (TIF)

S1 Table The associations between maternal education, preterm birth, small for gestational age, and infant mortality. (DOCX)

S2 Table The joint contribution of preterm birth and small for gestational age in explaining the association between maternal education and infant mortality according to birth year. (DOCX)

S3 Table The individual contribution of preterm birth and small for gestational age in explaining the association between maternal education and infant mortality according to birth year. (DOCX)

S4 Table The contribution of preterm birth in explaining the association between maternal education and mortality among infants that are not small for gestational age. (DOCX)

S5 Table The contribution of small for gestational age in explaining the association between maternal education and mortality among term-born infants. (DOCX)

S6 Table The contribution of preterm birth and small for gestational age in explaining the association between maternal education and infant mortality due to certain conditions originating in the perinatal period. (DOCX)

S7 Table The contribution of preterm birth and small for gestational age in explaining the association between maternal education and infant mortality due to congenital malformations. (DOCX)

S8 Table The contribution of preterm birth and small for gestational age in explaining the association between maternal education and infant mortality due to external causes. (DOCX)

S9 Table Sensitivity analyses of the influence of unmeasured mediator–outcome confounding. (DOCX)

S10 Table The contribution of preterm birth and small for gestational age in explaining the association between maternal education and infant mortality with an additional adjustment for maternal country of origin. (DOCX)

S11 Table The contribution of preterm birth and small for gestational age in explaining the association between maternal education and infant mortality using the traditional approach with and without mediators for mediation analysis. (DOCX)

S1 Text Analysis plan. (DOCX)

S2 Text A detailed description of the inverse probability weighting approach for causal mediation analysis. (DOCX)

## Abbreviations

CDE: controlled direct effect
IPMW: inverse-probability-of-mediators weight
IPTW: inverse-probability-of-treatment weight
LBW: low birth weight
MRR: mortality rate ratio
MSM: marginal structural model
PE: portion eliminated
PTB: preterm birth
SGA: small for gestational age
TE: total effect
