# Supplementary figures and images for: Mediating roles of preterm birth and restricted fetal growth in the relationship between maternal education and infant mortality: A Danish population-based cohort study
Source: PLoS Med. 2019 Jun 14;16(6):e1002831. doi: 10.1371/journal.pmed.1002831 (PMC6568398; doi:10.1371/journal.pmed.1002831)

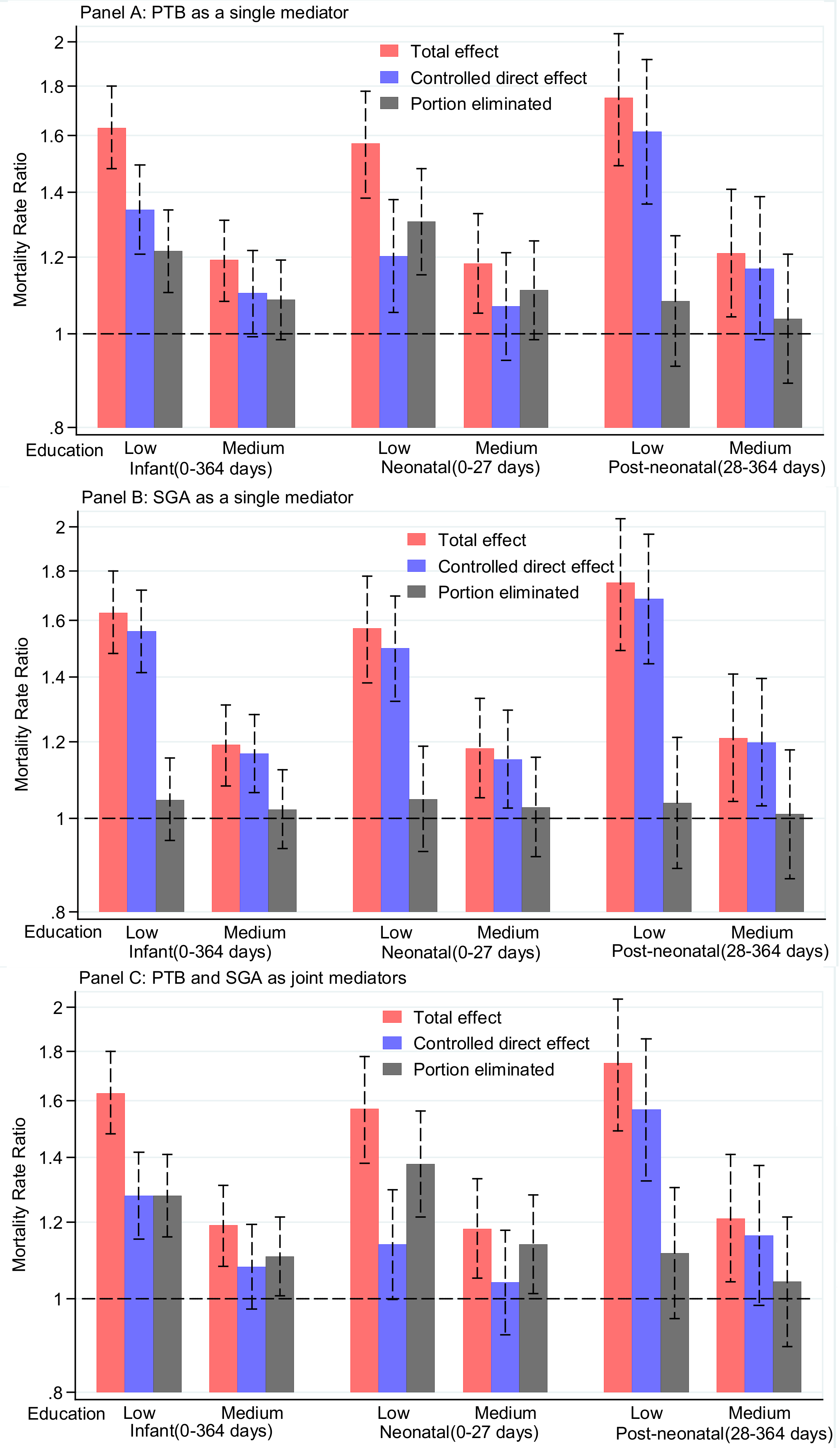

Supplement: S1 Fig — (TIF) [file pmed.1002831.s002.tif]
