# Supplementary material for: Mediating roles of preterm birth and restricted fetal growth in the relationship between maternal education and infant mortality: A Danish population-based cohort study
Source: PLoS Med. 2019 Jun 14;16(6):e1002831. doi: 10.1371/journal.pmed.1002831 (PMC6568398; doi:10.1371/journal.pmed.1002831)
Supplement: S1 Table — (DOCX) [file pmed.1002831.s003.docx]

**S1 Table. The associations between maternal education, preterm birth, small for gestational age, and infant mortality**

| **Dependent variable** | **Independent variable** | **Crude mortality rate ratio (95% CI)** | ***P* value** |
| --- | --- | --- | --- |
| **Infant death** | **Small for gestational age** |  |  |
|  | Yes | 2.56 (2.43-2.69) | 0.000 |
|  | No | 1.00(reference) |  |
| **Infant death** | **Preterm birth** |  |  |
|  | Yes | 15.56 (14.84-16.31) | 0.000 |
|  | No | 1.00(reference) |  |
|  |  | **Crude Odds ratio (95% CI)** | ***P* value** |
| **Small for gestational age** | **Education** |  |  |
|  | Low | 1.68 (1.66-1.70) | 0.000 |
|  | Medium | 1.22 (1.20-1.23) | 0.000 |
|  | High | 1.00(reference) |  |
| **Preterm birth** | **Education** |  |  |
|  | Low | 2.03 (1.98-2.08) | 0.000 |
|  | Medium | 1.32 (1.29-1.36) | 0.000 |
|  | High | 1.00(reference) |  |
