# Supplementary material for: Mediating roles of preterm birth and restricted fetal growth in the relationship between maternal education and infant mortality: A Danish population-based cohort study
Source: PLoS Med. 2019 Jun 14;16(6):e1002831. doi: 10.1371/journal.pmed.1002831 (PMC6568398; doi:10.1371/journal.pmed.1002831)
Supplement: S2 Table — (DOCX) [file pmed.1002831.s004.docx]

**S2 Table. The joint contribution of preterm birth and small for gestational age in explaining the association between maternal education and infant mortality according to birth year** ^a^

| **Birth year** | **Period** | **Education** | **No. of deaths** | **Rate/10^2^ pys** | **MRR_TE_** | ***P* value** | **MRR_CDE_** | ***P* value** | **MRR_PE_** | ***P* value** | **Proportion eliminated** |
| --- | --- | --- | --- | --- | --- | --- | --- | --- | --- | --- | --- |
| 1981-1985 | Infant | Low | 889 | 8.45 | 1.38 (1.10-1.72) | 0.005 | 1.05 (0.82-1.35) | 0.680 | 1.31 (1.05-1.63) | 0.019 | 86% |
|  | (< 1 year) | Medium | 555 | 6.37 | 1.13 (0.90-1.42) | 0.296 | 1.00 (0.77-1.28) | 0.981 | 1.13 (0.90-1.43) | 0.285 | - |
|  |  | High | 294 | 6.23 |  |  |  |  |  |  |  |
|  | Neonatal | Low | 496 | 63.38 | 1.14 (0.84-1.54) | 0.405 | 0.81 (0.58-1.13) | 0.217 | 1.40 (1.04-1.89) | 0.029 | - |
|  | (0-27 days) | Medium | 335 | 51.72 | 0.96 (0.70-1.31) | 0.795 | 0.84 (0.60-1.17) | 0.302 | 1.15 (0.84-1.56) | 0.386 | - |
|  |  | High | 186 | 53.04 |  |  |  |  |  |  |  |
|  | Postneonatal | Low | 393 | 4.04 | 1.92 (1.50-2.45) | 0.000 | 1.70 (1.32-2.20) | 0.000 | 1.13 (0.88-1.44) | 0.345 | 23% |
|  | (28-364 days) | Medium | 220 | 2.73 | 1.51 (1.17-1.97) | 0.002 | 1.44 (1.10-1.88) | 0.009 | 1.06 (0.81-1.37) | 0.686 | 15% |
|  |  | High | 108 | 2.47 |  |  |  |  |  |  |  |
| 1986-1990 | Infant | Low | 948 | 9.30 | 1.39 (1.14-1.71) | 0.001 | 1.08 (0.87-1.35) | 0.464 | 1.29 (1.05-1.58) | 0.016 | 79% |
|  | (< 1 year) | Medium | 756 | 6.37 | 1.11 (0.91-1.37) | 0.299 | 0.97 (0.78-1.21) | 0.774 | 1.15 (0.94-1.41) | 0.178 | - |
|  |  | High | 331 | 5.80 |  |  |  |  |  |  |  |
|  | Neonatal | Low | 493 | 64.99 | 1.42 (1.10-1.84) | 0.007 | 0.98 (0.75-1.29) | 0.908 | 1.44 (1.12-1.86) | 0.005 | - |
|  | (0-27 days) | Medium | 471 | 53.41 | 1.21 (0.94-1.55) | 0.146 | 0.98 (0.75-1.29) | 0.898 | 1.23 (0.95-1.58) | 0.112 | - |
|  |  | High | 198 | 46.66 |  |  |  |  |  |  |  |
|  | Postneonatal | Low | 455 | 4.82 | 1.36 (0.98-1.88) | 0.067 | 1.25 (0.87-1.79) | 0.232 | 1.09 (0.78-1.51) | 0.613 | 31% |
|  | (28-364 days) | Medium | 285 | 2.59 | 1.00 (0.71-1.40) | 0.989 | 0.95 (0.66-1.38) | 0.797 | 1.05 (0.75-1.47) | 0.788 | - |
|  |  | High | 133 | 2.52 |  |  |  |  |  |  |  |
| 1991-1995 | Infant | Low | 624 | 6.72 | 1.74 (1.40-2.15) | 0.000 | 1.37 (1.11-1.68) | 0.004 | 1.27 (1.03-1.57) | 0.027 | 50% |
|  | (< 1 year) | Medium | 649 | 4.15 | 1.16 (0.95-1.43) | 0.152 | 1.11 (0.91-1.36) | 0.311 | 1.05 (0.85-1.28) | 0.673 | 31% |
|  |  | High | 261 | 3.84 |  |  |  |  |  |  |  |
|  | Neonatal | Low | 362 | 52.46 | 1.79 (1.41-2.26) | 0.000 | 1.29 (1.01-1.65) | 0.045 | 1.39 (1.10-1.75) | 0.006 | 63% |
|  | (0-27 days) | Medium | 423 | 36.48 | 1.23 (0.99-1.54) | 0.061 | 1.13 (0.89-1.44) | 0.306 | 1.09 (0.87-1.36) | 0.443 | 44% |
|  |  | High | 178 | 35.29 |  |  |  |  |  |  |  |
|  | Postneonatal | Low | 262 | 3.05 | 1.66 (1.12-2.47) | 0.013 | 1.49 (1.03-2.17) | 0.035 | 1.11 (0.75-1.65) | 0.606 | 25% |
|  | (28-364 days) | Medium | 226 | 1.56 | 1.06 (0.71-1.57) | 0.789 | 1.07 (0.74-1.56) | 0.708 | 0.98 (0.66-1.46) | 0.931 | - |
|  |  | High | 83 | 1.32 |  |  |  |  |  |  |  |
| 1996-2000 | Infant | Low | 370 | 5.11 | 1.50 (1.18-1.90) | 0.001 | 1.10 (0.87-1.39) | 0.432 | 1.36 (1.08-1.73) | 0.010 | 80% |
|  | (< 1 year) | Medium | 555 | 3.58 | 1.05 (0.85-1.29) | 0.680 | 0.91 (0.74-1.13) | 0.410 | 1.14 (0.92-1.42) | 0.215 | - |
|  |  | High | 242 | 3.16 |  |  |  |  |  |  |  |
|  | Neonatal | Low | 236 | 43.91 | 1.66 (1.27-2.17) | 0.000 | 1.14 (0.86-1.50) | 0.359 | 1.46 (1.12-1.91) | 0.005 | 79% |
|  | (0-27 days) | Medium | 396 | 34.40 | 1.21 (0.96-1.52) | 0.102 | 1.04 (0.81-1.32) | 0.768 | 1.17 (0.93-1.47) | 0.186 | 82% |
|  |  | High | 172 | 30.21 |  |  |  |  |  |  |  |
|  | Postneonatal | Low | 134 | 2.00 | 1.25 (0.81-1.93) | 0.313 | 1.03 (0.67-1.57) | 0.899 | 1.22 (0.79-1.88) | 0.376 | 89% |
|  | (28-364 days) | Medium | 159 | 1.11 | 0.79 (0.52-1.20) | 0.269 | 0.71 (0.48-1.05) | 0.086 | 1.12 (0.74-1.69) | 0.590 | - |
|  |  | High | 70 | 0.99 |  |  |  |  |  |  |  |
| 2001-2005 | Infant | Low | 283 | 4.91 | 1.98 (1.48-2.65) | 0.000 | 1.42 (1.08-1.87) | 0.011 | 1.39 (1.04-1.86) | 0.027 | 57% |
|  | (< 1 year) | Medium | 435 | 3.01 | 1.29 (1.00-1.67) | 0.052 | 1.07 (0.84-1.35) | 0.598 | 1.21 (0.94-1.57) | 0.146 | 77% |
|  |  | High | 234 | 2.42 |  |  |  |  |  |  |  |
|  | Neonatal | Low | 170 | 39.64 | 1.54 (1.07-2.22) | 0.020 | 1.07 (0.77-1.50) | 0.688 | 1.44 (1.00-2.07) | 0.050 | 87% |
|  | (0-27 days) | Medium | 299 | 27.86 | 1.17 (0.85-1.61) | 0.347 | 0.93 (0.70-1.24) | 0.623 | 1.26 (0.91-1.73) | 0.166 | - |
|  |  | High | 175 | 24.32 |  |  |  |  |  |  |  |
|  | Postneonatal | Low | 113 | 2.12 | 3.45 (2.34-5.09) | 0.000 | 2.74 (1.83-4.08) | 0.000 | 1.26 (0.86-1.86) | 0.240 | 29% |
|  | (28-364 days) | Medium | 136 | 1.02 | 1.72 (1.24-2.37) | 0.001 | 1.58 (1.13-2.22) | 0.008 | 1.09 (0.79-1.50) | 0.617 | 19% |
|  |  | High | 59 | 0.66 |  |  |  |  |  |  |  |
| 2006-2010 | Infant | Low | 159 | 3.26 | 1.73 (1.28-2.35) | 0.000 | 1.33 (0.97-1.83) | 0.079 | 1.30 (0.96-1.76) | 0.085 | 55% |
|  | (< 1 year) | Medium | 297 | 2.35 | 1.47 (1.20-1.79) | 0.000 | 1.36 (1.09-1.68) | 0.005 | 1.08 (0.89-1.32) | 0.442 | 24% |
|  |  | High | 193 | 1.65 |  |  |  |  |  |  |  |
|  | Neonatal | Low | 102 | 28.13 | 1.66 (1.15-2.39) | 0.007 | 1.19 (0.81-1.73) | 0.374 | 1.40 (0.97-2.02) | 0.072 | 72% |
|  | (0-27 days) | Medium | 205 | 21.83 | 1.41 (1.11-1.80) | 0.005 | 1.28 (0.98-1.66) | 0.067 | 1.11 (0.87-1.41) | 0.409 | 33% |
|  |  | High | 135 | 15.55 |  |  |  |  |  |  |  |
|  | Postneonatal | Low | 57 | 1.26 | 1.91 (1.12-3.25) | 0.017 | 1.72 (0.97-3.04) | 0.061 | 1.11 (0.65-1.89) | 0.696 | 21% |
|  | (28-364 days) | Medium | 92 | 0.79 | 1.60 (1.13-2.25) | 0.008 | 1.57 (1.10-2.26) | 0.014 | 1.01 (0.72-1.43) | 0.935 | 4% |
|  |  | High | 58 | 0.54 |  |  |  |  |  |  |  |
| 2011-2015 | Infant | Low | 125 | 3.36 | 2.53 (1.83-3.49) | 0.000 | 1.68 (1.22-2.31) | 0.002 | 1.50 (1.09-2.08) | 0.014 | 56% |
|  | (< 1 year) | Medium | 205 | 2.06 | 1.71 (1.37-2.13) | 0.000 | 1.46 (1.15-1.86) | 0.002 | 1.17 (0.94-1.45) | 0.170 | 35% |
|  |  | High | 158 | 1.38 |  |  |  |  |  |  |  |
|  | Neonatal | Low | 85 | 30.76 | 2.76 (1.87-4.07) | 0.000 | 1.65 (1.12-2.43) | 0.011 | 1.67 (1.13-2.46) | 0.010 | 63% |
|  | (0-27 days) | Medium | 139 | 18.85 | 1.66 (1.27-2.16) | 0.000 | 1.32 (0.99-1.76) | 0.056 | 1.25 (0.96-1.63) | 0.094 | 51% |
|  |  | High | 114 | 13.45 |  |  |  |  |  |  |  |
|  | Postneonatal | Low | 40 | 1.16 | 1.94 (1.15-3.25) | 0.013 | 1.74 (1.01-3.00) | 0.045 | 1.11 (0.66-1.86) | 0.692 | 21% |
|  | (28-364 days) | Medium | 66 | 0.72 | 1.84 (1.23-2.74) | 0.003 | 1.87 (1.22-2.86) | 0.004 | 0.98 (0.66-1.47) | 0.923 | - |
|  |  | High | 44 | 0.42 |  |  |  |  |  |  |  |

^a^ Pys, person-years; TE, total effect; CDE, controlled direct effect; PE, portion eliminated; MRR, mortality rate ratio; proportion eliminated: = (MRR_TE_ – MRR_CDE_)/(MRR_TE_-1); proportion eliminated is only presented if the MRRs of CDE and PE were in the same direction.
