# Supplementary material for: Mediating roles of preterm birth and restricted fetal growth in the relationship between maternal education and infant mortality: A Danish population-based cohort study
Source: PLoS Med. 2019 Jun 14;16(6):e1002831. doi: 10.1371/journal.pmed.1002831 (PMC6568398; doi:10.1371/journal.pmed.1002831)
Supplement: S3 Table — (DOCX) [file pmed.1002831.s005.docx]

**S3 Table. The individual contribution of preterm birth and small for gestational age in explaining the association between maternal education and infant mortality according to birth year** ^a^

|  |  | | |  | | |  | | **Preterm birth** | | | | |  | |  | |  | |  | | | | **Small for gestational age** | | | |  | |
| --- | --- | --- | --- | --- | --- | --- | --- | --- | --- | --- | --- | --- | --- | --- | --- | --- | --- | --- | --- | --- | --- | --- | --- | --- | --- | --- | --- | --- | --- |
| **Birth year** | | **Period** | | | **Education** | | | **MRR_CDE_** | | **P value** | **MRR_PE_** | **P value** | **Proportion eliminated** | | **MRR_CDE_** | | | | **P value** | | | **MRR_PE_** | | | | **P value** | **Proportion eliminated** | |  |
| 1981-1985 | | Infant | | | Low | | | 1.11 (0.85-1.43) | | 0.445 | 1.24 (1.00-1.55) | 0.055 | 72% | | 1.29 (1.04-1.60) | | | | 0.021 | | | 1.07 (0.85-1.33) | | | | 0.567 | 23% | |  |
|  | | (< 1 year) | | | Medium | | | 1.02 (0.78-1.32) | | 0.903 | 1.11 (0.88-1.40) | 0.366 | 87% | | 1.09 (0.87-1.36) | | | | 0.451 | | | 1.04 (0.82-1.30) | | | | 0.756 | 31% | |  |
|  | |  | | | High | | |  | |  |  |  |  | |  | | | |  | | |  | | | |  |  | |  |
|  | | Neonatal | | | Low | | | 0.83 (0.59-1.18) | | 0.305 | 1.36 (1.01-1.84) | 0.045 | - | | 1.08 (0.80-1.44) | | | | 0.625 | | | 1.06 (0.78-1.43) | | | | 0.718 | 45% | |  |
|  | | (0-27 days) | | | Medium | | | 0.84 (0.59-1.20) | | 0.344 | 1.14 (0.84-1.55) | 0.414 | - | | 0.92 (0.69-1.25) | | | | 0.610 | | | 1.04 (0.76-1.41) | | | | 0.813 | - | |  |
|  | |  | | | High | | |  | |  |  |  |  | |  | | | |  | | |  | | | |  |  | |  |
|  | | Postneonatal | | | Low | | | 1.85 (1.45-2.37) | | 0.000 | 1.03 (0.81-1.32) | 0.784 | 7% | | 1.76 (1.38-2.26) | | | | 0.000 | | | 1.09 (0.85-1.39) | | | | 0.505 | 17% | |  |
|  | | (28-364 days) | | | Medium | | | 1.50 (1.15-1.95) | | 0.002 | 1.01 (0.78-1.31) | 0.941 | 3% | | 1.45 (1.11-1.89) | | | | 0.006 | | | 1.04 (0.80-1.35) | | | | 0.752 | 12% | |  |
|  | |  | | | High | | |  | |  |  |  |  | |  | | | |  | | |  | | | |  |  | |  |
| 1986-1990 | | Infant | | | Low | | | 1.15 (0.93-1.44) | | 0.198 | 1.21 (0.99-1.48) | 0.069 | 61% | | 1.31 (1.07-1.60) | | | | 0.009 | | | 1.07 (0.87-1.31) | | | | 0.527 | 22% | |  |
|  | | (< 1 year) | | | Medium | | | 1.01 (0.81-1.26) | | 0.945 | 1.11 (0.90-1.36) | 0.335 | 93% | | 1.07 (0.88-1.31) | | | | 0.496 | | | 1.04 (0.85-1.28) | | | | 0.715 | 36% | |  |
|  | |  | | | High | | |  | |  |  |  |  | |  | | | |  | | |  | | | |  |  | |  |
|  | | Neonatal | | | Low | | | 1.08 (0.83-1.41) | | 0.563 | 1.31 (1.02-1.70) | 0.036 | 81% | | 1.28 (0.99-1.65) | | | | 0.062 | | | 1.11 (0.86-1.44) | | | | 0.415 | 34% | |  |
|  | | (0-27 days) | | | Medium | | | 1.06 (0.81-1.37) | | 0.688 | 1.14 (0.89-1.47) | 0.301 | 73% | | 1.12 (0.87-1.45) | | | | 0.371 | | | 1.07 (0.83-1.38) | | | | 0.577 | 41% | |  |
|  | |  | | | High | | |  | |  |  |  |  | |  | | | |  | | |  | | | |  |  | |  |
|  | | Postneonatal | | | Low | | | 1.25 (0.86-1.82) | | 0.237 | 1.08 (0.78-1.50) | 0.630 | 29% | | 1.34 (0.98-1.85) | | | | 0.070 | | | 1.01 (0.73-1.40) | | | | 0.950 | 4% | |  |
|  | | (28-364 days) | | | Medium | | | 0.94 (0.64-1.38) | | 0.754 | 1.06 (0.76-1.49) | 0.733 | - | | 1.00 (0.72-1.40) | | | | 0.984 | | | 0.99 (0.71-1.39) | | | | 0.973 | - | |  |
|  | |  | | | High | | |  | |  |  |  |  | |  | | | |  | | |  | | | |  |  | |  |
| 1991-1995 | | Infant | | | Low | | | 1.43 (1.15-1.78) | | 0.001 | 1.21 (0.98-1.50) | 0.074 | 42% | | 1.65 (1.35-2.03) | | | | 0.000 | | | 1.05 (0.85-1.30) | | | | 0.654 | 11% | |  |
|  | | (< 1 year) | | | Medium | | | 1.09 (0.89-1.35) | | 0.411 | 1.06 (0.87-1.31) | 0.555 | 43% | | 1.17 (0.96-1.43) | | | | 0.118 | | | 0.99 (0.81-1.22) | | | | 0.944 | - | |  |
|  | |  | | | High | | |  | |  |  |  |  | |  | | | |  | | |  | | | |  |  | |  |
|  | | Neonatal | | | Low | | | 1.35 (1.05-1.73) | | 0.017 | 1.32 (1.05-1.67) | 0.019 | 55% | | 1.69 (1.34-2.14) | | | | 0.000 | | | 1.06 (0.84-1.33) | | | | 0.650 | 12% | |  |
|  | | (0-27 days) | | | Medium | | | 1.12 (0.88-1.41) | | 0.349 | 1.10 (0.89-1.38) | 0.381 | 49% | | 1.24 (0.99-1.54) | | | | 0.057 | | | 1.00 (0.80-1.24) | | | | 0.972 | - | |  |
|  | |  | | | High | | |  | |  |  |  |  | |  | | | |  | | |  | | | |  |  | |  |
|  | | Postneonatal | | | Low | | | 1.56 (1.05-2.33) | | 0.030 | 1.06 (0.71-1.58) | 0.765 | 15% | | 1.59 (1.10-2.31) | | | | 0.014 | | | 1.04 (0.70-1.55) | | | | 0.843 | 10% | |  |
|  | | (28-364 days) | | | Medium | | | 1.05 (0.70-1.56) | | 0.817 | 1.01 (0.68-1.49) | 0.973 | 13% | | 1.07 (0.74-1.54) | | | | 0.724 | | | 0.99 (0.67-1.47) | | | | 0.952 | - | |  |
|  | |  | | | High | | |  | |  |  |  |  | |  | | | |  | | |  | | | |  |  | |  |
| 1996-2000 | | Infant | | | Low | | | 1.23 (0.98-1.55) | | 0.080 | 1.22 (0.96-1.54) | 0.100 | 54% | | 1.33 (1.05-1.68) | | | | 0.016 | | | 1.13 (0.89-1.43) | | | | 0.322 | 34% | |  |
|  | | (< 1 year) | | | Medium | | | 0.97 (0.79-1.20) | | 0.778 | 1.08 (0.87-1.33) | 0.492 | - | | 0.98 (0.80-1.21) | | | | 0.858 | | | 1.07 (0.86-1.32) | | | | 0.556 | - | |  |
|  | |  | | | High | | |  | |  |  |  |  | |  | | | |  | | |  | | | |  |  | |  |
|  | | Neonatal | | | Low | | | 1.26 (0.97-1.65) | | 0.085 | 1.31 (1.01-1.72) | 0.044 | 60% | | 1.49 (1.15-1.94) | | | | 0.003 | | | 1.12 (0.85-1.46) | | | | 0.421 | 26% | |  |
|  | | (0-27 days) | | | Medium | | | 1.09 (0.86-1.36) | | 0.481 | 1.12 (0.89-1.40) | 0.350 | 60% | | 1.16 (0.92-1.45) | | | | 0.210 | | | 1.05 (0.83-1.32) | | | | 0.694 | 26% | |  |
|  | |  | | | High | | |  | |  |  |  |  | |  | | | |  | | |  | | | |  |  | |  |
|  | | Postneonatal | | | Low | | | 1.17 (0.76-1.79) | | 0.481 | 1.07 (0.69-1.66) | 0.753 | 34% | | 1.10 (0.72-1.68) | | | | 0.667 | | | 1.14 (0.74-1.76) | | | | 0.558 | 61% | |  |
|  | | (28-364 days) | | | Medium | | | 0.77 (0.51-1.16) | | 0.217 | 1.02 (0.68-1.55) | 0.907 | - | | 0.72 (0.48-1.08) | | | | 0.113 | | | 1.09 (0.72-1.66) | | | | 0.668 | - | |  |
|  | |  | | | High | | |  | |  |  |  |  | |  | | | |  | | |  | | | |  |  | |  |
| 2001-2005 | | Infant | | | Low | | | 1.56 (1.18-2.06) | | 0.002 | 1.27 (0.95-1.70) | 0.111 | 43% | | 1.80 (1.36-2.39) | | | | 0.000 | | | 1.10 (0.82-1.47) | | | | 0.535 | 18% | |  |
|  | | (< 1 year) | | | Medium | | | 1.15 (0.90-1.48) | | 0.267 | 1.12 (0.87-1.45) | 0.386 | 48% | | 1.21 (0.95-1.55) | | | | 0.120 | | | 1.07 (0.82-1.38) | | | | 0.630 | 27% | |  |
|  | |  | | | High | | |  | |  |  |  |  | |  | | | |  | | |  | | | |  |  | |  |
|  | | Neonatal | | | Low | | | 1.16 (0.82-1.64) | | 0.412 | 1.33 (0.93-1.92) | 0.121 | 71% | | 1.45 (1.02-2.05) | | | | 0.039 | | | 1.07 (0.74-1.54) | | | | 0.728 | 18% | |  |
|  | | (0-27 days) | | | Medium | | | 1.01 (0.74-1.37) | | 0.973 | 1.16 (0.84-1.60) | 0.363 | 97% | | 1.10 (0.81-1.48) | | | | 0.547 | | | 1.06 (0.77-1.47) | | | | 0.704 | 42% | |  |
|  | |  | | | High | | |  | |  |  |  |  | |  | | | |  | | |  | | | |  |  | |  |
|  | | Postneonatal | | | Low | | | 3.06 (2.09-4.48) | | 0.000 | 1.13 (0.77-1.66) | 0.543 | 16% | | 2.99 (2.01-4.46) | | | | 0.000 | | | 1.15 (0.78-1.70) | | | | 0.471 | 19% | |  |
|  | | (28-364 days) | | | Medium | | | 1.70 (1.23-2.35) | | 0.001 | 1.01 (0.73-1.39) | 0.950 | 2% | | 1.60 (1.14-2.23) | | | | 0.006 | | | 1.07 (0.78-1.48) | | | | 0.665 | 16% | |  |
|  | |  | | | High | | |  | |  |  |  |  | |  | | | |  | | |  | | | |  |  | |  |
| 2006-2010 | | Infant | | | Low | | | 1.37 (1.01-1.87) | | 0.042 | 1.26 (0.93-1.71) | 0.130 | 49% | | 1.71 (1.25-2.34) | | | | 0.001 | | | 1.02 (0.75-1.37) | | | | 0.919 | 4% | |  |
|  | | (< 1 year) | | | Medium | | | 1.37 (1.11-1.68) | | 0.003 | 1.07 (0.88-1.30) | 0.502 | 21% | | 1.45 (1.19-1.78) | | | | 0.000 | | | 1.01 (0.83-1.23) | | | | 0.936 | 3% | |  |
|  | |  | | | High | | |  | |  |  |  |  | |  | | | |  | | |  | | | |  |  | |  |
|  | | Neonatal | | | Low | | | 1.19 (0.83-1.71) | | 0.336 | 1.39 (0.96-2.00) | 0.077 | 71% | | 1.69 (1.15-2.48) | | | | 0.007 | | | 0.98 (0.68-1.42) | | | | 0.923 | - | |  |
|  | | (0-27 days) | | | Medium | | | 1.29 (1.00-1.65) | | 0.050 | 1.10 (0.86-1.40) | 0.445 | 31% | | 1.41 (1.11-1.81) | | | | 0.006 | | | 1.00 (0.78-1.27) | | | | 0.989 | - | |  |
|  | |  | | | High | | |  | |  |  |  |  | |  | | | |  | | |  | | | |  |  | |  |
|  | | Postneonatal | | | Low | | | 1.87 (1.08-3.24) | | 0.024 | 1.02 (0.60-1.74) | 0.940 | 4% | | 1.74 (1.00-3.03) | | | | 0.049 | | | 1.10 (0.64-1.86) | | | | 0.735 | 18% | |  |
|  | | (28-364 days) | | | Medium | | | 1.60 (1.13-2.27) | | 0.008 | 1.00 (0.71-1.41) | 0.981 | - | | 1.54 (1.08-2.20) | | | | 0.017 | | | 1.03 (0.73-1.46) | | | | 0.850 | 9% | |  |
|  | |  | | | High | | |  | |  |  |  |  | |  | | | |  | | |  | | | |  |  | |  |
| 2011-2015 | | Infant | | | Low | | | 1.82 (1.32-2.51) | | 0.000 | 1.39 (1.00-1.92) | 0.049 | 46% | | 2.40 (1.72-3.35) | | | | 0.000 | | | 1.05 (0.76-1.45) | | | | 0.764 | 8% | |  |
|  | | (< 1 year) | | | Medium | | | 1.48 (1.18-1.86) | | 0.001 | 1.15 (0.93-1.44) | 0.204 | 32% | | 1.65 (1.32-2.07) | | | | 0.000 | | | 1.03 (0.83-1.29) | | | | 0.778 | 8% | |  |
|  | |  | | | High | | |  | |  |  |  |  | |  | | | |  | | |  | | | |  |  | |  |
|  | | Neonatal | | | Low | | | 1.80 (1.22-2.66) | | 0.003 | 1.53 (1.03-2.25) | 0.033 | 54% | | 2.64 (1.77-3.92) | | | | 0.000 | | | 1.04 (0.71-1.54) | | | | 0.828 | 7% | |  |
|  | | (0-27 days) | | | Medium | | | 1.35 (1.02-1.78) | | 0.034 | 1.23 (0.94-1.60) | 0.127 | 47% | | 1.59 (1.22-2.08) | | | | 0.001 | | | 1.04 (0.80-1.35) | | | | 0.766 | 10% | |  |
|  | |  | | | High | | |  | |  |  |  |  | |  | | | |  | | |  | | | |  |  | |  |
|  | | Postneonatal | | | Low | | | 1.87 (1.10-3.17) | | 0.020 | 1.03 (0.62-1.74) | 0.897 | 7% | | 1.80 (1.06-3.08) | | | | 0.031 | | | 1.07 (0.64-1.80) | | | | 0.791 | 14% | |  |
|  | | (28-364 days) | | | Medium | | | 1.87 (1.25-2.82) | | 0.003 | 0.98 (0.66-1.46) | 0.920 | - | | 1.81 (1.19-2.75) | | | | 0.006 | | | 1.01 (0.68-1.52) | | | | 0.945 | 3% | |  |
|  | | |  | | | High | |  | |  |  |  |  | | | |  | | | |  | |  | |  | |  | | |

^a^ Pys, person-years; TE, total effect; CDE, controlled direct effect; PE, portion eliminated; MRR, mortality rate ratio; proportion eliminated: = (MRR_TE_ – MRR_CDE_)/(MRR_TE_-1); proportion eliminated is only presented if the MRRs of CDE and PE were in the same direction.
