# Supplementary material for: Mediating roles of preterm birth and restricted fetal growth in the relationship between maternal education and infant mortality: A Danish population-based cohort study
Source: PLoS Med. 2019 Jun 14;16(6):e1002831. doi: 10.1371/journal.pmed.1002831 (PMC6568398; doi:10.1371/journal.pmed.1002831)
Supplement: S4 Table — (DOCX) [file pmed.1002831.s006.docx]

**S4 Table. The contribution of preterm birth in explaining the association between maternal education and mortality among non-SGA infants that are not small for gestational age ^a^**

| **Period** | **Education** | **No. of deaths** | **Rate/10^2^ pys** | **MRR_TE_** | ***P* value** | **MRR_CDE_** | ***P* value** | **MRR_PE_** | ***P* value** | **Proportion eliminated** |
| --- | --- | --- | --- | --- | --- | --- | --- | --- | --- | --- |
| Infant | Low | 2,660 | 5.88 | 1.65 (1.47-1.85) | 0.000 | 1.32 (1.17-1.49) | 0.000 | 1.25 (1.12-1.40) | 0.000 | 50% |
| (< 1 year) | Medium | 2,718 | 3.37 | 1.20 (1.08-1.33) | 0.001 | 1.10 (0.98-1.24) | 0.120 | 1.09 (0.98-1.21) | 0.113 | 50% |
|  | High | 1,357 | 2.55 | 1.00(reference) |  |  |  |  |  |  |
| Neonatal | Low | 1,514 | 45.02 | 1.58 (1.37-1.83) | 0.000 | 1.16 (1.00-1.36) | 0.053 | 1.36 (1.17-1.57) | 0.000 | 72% |
| (0-27 days) | Medium | 1,776 | 29.70 | 1.18 (1.03-1.35) | 0.018 | 1.05 (0.91-1.22) | 0.476 | 1.12 (0.97-1.28) | 0.112 | 69% |
|  | High | 924 | 23.37 | 1.00(reference) |  |  |  |  |  |  |
| Postneonatal | Low | 1,146 | 2.74 | 1.78 (1.49-2.12) | 0.000 | 1.63 (1.34-1.99) | 0.000 | 1.09 (0.91-1.30) | 0.348 | 19% |
| (28-364 days) | Medium | 942 | 1.26 | 1.23 (1.04-1.47) | 0.017 | 1.18 (0.97-1.44) | 0.090 | 1.04 (0.88-1.24) | 0.638 | 21% |
|  | High | 433 | 0.88 | 1.00(reference) |  |  |  |  |  |  |

^a^ Pys, person-years; TE, total effect; CDE, controlled direct effect; PE, portion eliminated; MRR, mortality rate ratio; proportion eliminated: = (MRR_TE_ – MRR_CDE_)/(MRR_TE_-1); proportion eliminated is only presented if the MRRs of CDE and PE were in the same direction.
