# Supplementary material for: Mediating roles of preterm birth and restricted fetal growth in the relationship between maternal education and infant mortality: A Danish population-based cohort study
Source: PLoS Med. 2019 Jun 14;16(6):e1002831. doi: 10.1371/journal.pmed.1002831 (PMC6568398; doi:10.1371/journal.pmed.1002831)
Supplement: S5 Table — (DOCX) [file pmed.1002831.s007.docx]

**S5 Table. The contribution of small for gestational age in explaining the association between maternal education and mortality among term-born infants ^a^**

| **Period** | **Education** | **No. of deaths** | **Rate/10^2^ pys** | **MRR_TE_** | ***P* value** | **MRR_CDE_** | ***P* value** | **MRR_PE_** | ***P* value** | **Proportion eliminated** |
| --- | --- | --- | --- | --- | --- | --- | --- | --- | --- | --- |
| Infant | Low | 1,907 | 3.89 | 1.51 (1.33-1.72) | 0.000 | 1.42 (1.25-1.62) | 0.000 | 1.06 (0.93-1.21) | 0.359 | 17% |
| (< 1 year) | Medium | 1,914 | 2.25 | 1.14 (1.01-1.29) | 0.035 | 1.11 (0.98-1.26) | 0.088 | 1.03 (0.91-1.16) | 0.670 | 21% |
|  | High | 954 | 1.71 | 1.00(reference) |  |  |  |  |  |  |
| Neonatal | Low | 727 | 19.98 | 1.39 (1.14-1.70) | 0.001 | 1.27 (1.05-1.55) | 0.016 | 1.09 (0.89-1.33) | 0.394 | 30% |
| (0-27 days) | Medium | 953 | 15.07 | 1.15 (0.96-1.39) | 0.135 | 1.11 (0.92-1.33) | 0.282 | 1.04 (0.86-1.26) | 0.661 | 31% |
|  | High | 480 | 11.59 | 1.00(reference) |  |  |  |  |  |  |
| Postneonatal | Low | 1,180 | 2.60 | 1.62 (1.38-1.91) | 0.000 | 1.56 (1.33-1.84) | 0.000 | 1.04 (0.88-1.22) | 0.670 | 9% |
| (28-364 days) | Medium | 961 | 1.22 | 1.13 (0.96-1.33) | 0.137 | 1.12 (0.95-1.31) | 0.182 | 1.01 (0.86-1.19) | 0.871 | 12% |
|  | High | 474 | 0.92 | 1.00(reference) |  |  |  |  |  |  |

^a^ Pys, person-years; TE, total effect; CDE, controlled direct effect; PE, portion eliminated; MRR, mortality rate ratio; proportion eliminated: = (MRR_TE_ – MRR_CDE_)/(MRR_TE_-1); proportion eliminated is only presented if the MRRs of CDE and PE were in the same direction; PTB, preterm birth; SGA; small for gestational age.
