# Supplementary material for: Mediating roles of preterm birth and restricted fetal growth in the relationship between maternal education and infant mortality: A Danish population-based cohort study
Source: PLoS Med. 2019 Jun 14;16(6):e1002831. doi: 10.1371/journal.pmed.1002831 (PMC6568398; doi:10.1371/journal.pmed.1002831)
Supplement: S6 Table — (DOCX) [file pmed.1002831.s008.docx]

**S6 Table. The contribution of preterm birth and small for gestational age in explaining the association between maternal education and infant mortality due to certain conditions originating in the perinatal period ^ab^**

| **Mediator** | **Period** | **Education** | **No. of deaths** | **Rate/10^2^ pys** | **MRR_TE_** | ***P* value** | **MRR_CDE_** | ***P* value** | **MRR_PE_** | ***P* value** | **Proportion eliminated** | |
| --- | --- | --- | --- | --- | --- | --- | --- | --- | --- | --- | --- | --- |
| PTB | Infant | Low | 1,121 | 2.17 | 1.77 (1.50-2.08) | 0.000 | 1.21 (1.00-1.45) | 0.044 | 1.46 (1.24-1.73) | 0.000 | 73% | |
|  | (< 1 year) | Medium | 1,309 | 1.48 | 1.28 (1.10-1.49) | 0.002 | 1.09 (0.92-1.30) | 0.329 | 1.17 (1.00-1.37) | 0.046 | 67% | |
|  |  | High | 639 | 1.11 | 1.00(reference) |  |  |  |  |  |  | |
|  | Neonatal | Low | 1,040 | 27.11 | 1.75 (1.47-2.08) | 0.000 | 1.19 (0.98-1.44) | 0.080 | 1.47 (1.24-1.75) | 0.000 | 75% | |
|  | (0-27 days) | Medium | 1,216 | 18.45 | 1.25 (1.06-1.47) | 0.007 | 1.06 (0.88-1.27) | 0.541 | 1.18 (1.00-1.39) | 0.043 | 77% | |
|  |  | High | 591 | 13.79 | 1.00(reference) |  |  |  |  |  |  | |
|  | Postneonatal | Low | 81 | 0.17 | 2.05 (1.31-3.22) | 0.002 | 1.54 (1.00-2.37) | 0.048 | 1.33 (0.85-2.09) | 0.212 | 49% | |
|  | (28-364 days) | Medium | 93 | 0.11 | 1.70 (1.16-2.48) | 0.007 | 1.70 (1.15-2.51) | 0.008 | 1.00 (0.68-1.47) | 0.999 | - | |
|  |  | High | 48 | 0.09 | 1.00(reference) |  |  |  |  |  |  | |
| SGA | Infant | Low | 1,121 | 2.17 | 1.77 (1.50-2.08) | 0.000 | 1.74 (1.48-2.05) | 0.000 | 1.01 (0.86-1.20) | 0.865 | 3% | |
|  | (< 1 year) | Medium | 1,309 | 1.48 | 1.28 (1.10-1.49) | 0.002 | 1.27 (1.09-1.48) | 0.002 | 1.01 (0.86-1.18) | 0.905 | 4% | |
|  |  | High | 639 | 1.11 | 1.00(reference) |  |  |  |  |  |  | |
|  | Neonatal | Low | 1,040 | 27.11 | 1.75 (1.47-2.08) | 0.000 | 1.72 (1.45-2.05) | 0.000 | 1.01 (0.85-1.21) | 0.883 | 3% | |
|  | (0-27 days) | Medium | 1,216 | 18.45 | 1.25 (1.06-1.47) | 0.007 | 1.24 (1.06-1.45) | 0.009 | 1.01 (0.86-1.19) | 0.897 | 5% | |
|  |  | High | 591 | 13.79 | 1.00(reference) |  |  |  |  |  |  | |
|  | Postneonatal | Low | 81 | 0.17 | 2.05 (1.31-3.22) | 0.002 | 2.05 (1.28-3.28) | 0.003 | 1.00 (0.64-1.57) | 0.995 | - | |
|  | (28-364 days) | Medium | 93 | 0.11 | 1.70 (1.16-2.48) | 0.007 | 1.74 (1.18-2.56) | 0.005 | 0.98 (0.67-1.43) | 0.902 | - | |
|  |  | High | 48 | 0.09 | 1.00(reference) |  |  |  |  |  |  | |
| PTB | Infant | Low | 1,121 | 2.17 | 1.77 (1.50-2.08) | 0.000 | 1.19 (0.99-1.42) | 0.061 | 1.49 (1.26-1.76) | 0.000 | 76% | |
| and | (< 1 year) | Medium | 1,309 | 1.48 | 1.28 (1.10-1.49) | 0.002 | 1.09 (0.91-1.29) | 0.347 | 1.18 (1.01-1.38) | 0.038 | 69% | |
| SGA |  | High | 639 | 1.11 | 1.00(reference) |  |  |  |  |  |  | |
|  | Neonatal | Low | 1,040 | 27.11 | 1.75 (1.47-2.08) | 0.000 | 1.17 (0.97-1.41) | 0.106 | 1.50 (1.26-1.78) | 0.000 | 78% | |
|  | (0-27 days) | Medium | 1,216 | 18.45 | 1.25 (1.06-1.47) | 0.007 | 1.05 (0.88-1.26) | 0.583 | 1.19 (1.01-1.40) | 0.035 | 80% | |
|  |  | High | 591 | 13.79 | 1.00(reference) |  |  |  |  |  |  | |
|  | Postneonatal | Low | 81 | 0.17 | 2.05 (1.31-3.22) | 0.002 | 1.55 (1.00-2.40) | 0.050 | 1.32 (0.84-2.07) | 0.222 | 48% | |
|  | (28-364 days) | Medium | 93 | 0.11 | 1.70 (1.16-2.48) | 0.007 | 1.75 (1.18-2.61) | 0.006 | 0.97 (0.66-1.42) | 0.866 | - | |
|  |  | High | 48 | 0.09 | 1.00(reference) |  |  |  |  |  | |  |

^a^ Deaths due to certain conditions originating in the perinatal period: ICD-8 codes 760-779 and ICD-10 codes P00-P96.

^b^ Pys, person-years; TE, total effect; CDE, controlled direct effect; PE, portion eliminated; MRR, mortality rate ratio; proportion eliminated: = (MRR_TE_ – MRR_CDE_)/(MRR_TE_-1); proportion eliminated is only presented if the MRRs of CDE and PE were in the same direction; PTB, preterm birth; SGA, small for gestational age.
