# Supplementary material for: Mediating roles of preterm birth and restricted fetal growth in the relationship between maternal education and infant mortality: A Danish population-based cohort study
Source: PLoS Med. 2019 Jun 14;16(6):e1002831. doi: 10.1371/journal.pmed.1002831 (PMC6568398; doi:10.1371/journal.pmed.1002831)
Supplement: S7 Table — (DOCX) [file pmed.1002831.s009.docx]

**S7 Table. The contribution of preterm birth and small for gestational age in explaining the association between maternal education and infant mortality due to congenital malformations ^ab^**

| **Mediator** | **Period** | **Education** | **No. of death** | **Rate/10^2^ pys** | **MRR_TE_** | ***P* value** | **MRR_CDE_** | ***P* value** | **MRR_PE_** | ***P* value** | **Proportion eliminated** | |
| --- | --- | --- | --- | --- | --- | --- | --- | --- | --- | --- | --- | --- |
| PTB | Infant | Low | 1,059 | 2.05 | 1.33 (1.13-1.56) | 0.001 | 1.21 (1.02-1.43) | 0.025 | 1.10 (0.93-1.29) | 0.262 | 36% | |
|  | (< 1 year) | Medium | 1,216 | 1.37 | 1.07 (0.91-1.25) | 0.421 | 1.03 (0.88-1.20) | 0.736 | 1.04 (0.89-1.21) | 0.643 | 58% | |
|  |  | High | 638 | 1.11 | 1.00(reference) |  |  |  |  |  |  | |
|  | Neonatal | Low | 701 | 18.27 | 1.27 (1.03-1.56) | 0.022 | 1.15 (0.93-1.42) | 0.187 | 1.11 (0.90-1.36) | 0.339 | 45% | |
|  | (0-27 days) | Medium | 819 | 12.43 | 1.06 (0.87-1.30) | 0.538 | 1.03 (0.85-1.26) | 0.733 | 1.03 (0.84-1.25) | 0.783 | 46% | |
|  |  | High | 436 | 10.18 | 1.00(reference) |  |  |  |  |  |  | |
|  | Postneonatal | Low | 358 | 0.75 | 1.44 (1.11-1.88) | 0.006 | 1.34 (1.01-1.76) | 0.040 | 1.08 (0.83-1.41) | 0.559 | 24% | |
|  | (28-364 days) | Medium | 397 | 0.48 | 1.07 (0.83-1.38) | 0.596 | 1.02 (0.78-1.33) | 0.894 | 1.05 (0.82-1.35) | 0.697 | 74% | |
|  |  | High | 202 | 0.38 | 1.00(reference) |  |  |  |  |  |  | |
| SGA | Infant | Low | 1,059 | 2.05 | 1.33 (1.13-1.56) | 0.001 | 1.20 (1.02-1.41) | 0.030 | 1.11 (0.94-1.31) | 0.212 | 40% | |
|  | (< 1 year) | Medium | 1,216 | 1.37 | 1.07 (0.91-1.25) | 0.421 | 1.02 (0.87-1.19) | 0.838 | 1.05 (0.90-1.23) | 0.547 | 75% | |
|  |  | High | 638 | 1.11 | 1.00(reference) |  |  |  |  |  |  | |
|  | Neonatal | Low | 701 | 18.27 | 1.27 (1.03-1.56) | 0.022 | 1.15 (0.94-1.41) | 0.182 | 1.11 (0.90-1.36) | 0.334 | 45% | |
|  | (0-27 days) | Medium | 819 | 12.43 | 1.06 (0.87-1.30) | 0.538 | 1.02 (0.84-1.24) | 0.864 | 1.05 (0.86-1.27) | 0.655 | 73% | |
|  |  | High | 436 | 10.18 | 1.00(reference) |  |  |  |  |  |  | |
|  | Postneonatal | Low | 358 | 0.75 | 1.44 (1.11-1.88) | 0.006 | 1.31 (1.00-1.71) | 0.051 | 1.11 (0.85-1.44) | 0.452 | 31% | |
|  | (28-364 days) | Medium | 397 | 0.48 | 1.07 (0.83-1.38) | 0.596 | 1.01 (0.78-1.31) | 0.916 | 1.06 (0.82-1.36) | 0.672 | 80% | |
|  |  | High | 202 | 0.38 | 1.00(reference) |  |  |  |  |  |  | |
| PTB | Infant | Low | 1,059 | 2.05 | 1.33 (1.13-1.56) | 0.001 | 1.07 (0.91-1.26) | 0.405 | 1.24 (1.05-1.46) | 0.010 | 78% | |
| and | (< 1 year) | Medium | 1,216 | 1.37 | 1.07 (0.91-1.25) | 0.421 | 0.97 (0.83-1.14) | 0.720 | 1.10 (0.94-1.28) | 0.244 | - | |
| SGA |  | High | 638 | 1.11 | 1.00(reference) |  |  |  |  |  |  | |
|  | Neonatal | Low | 701 | 18.27 | 1.27 (1.03-1.56) | 0.022 | 1.01 (0.83-1.24) | 0.905 | 1.26 (1.02-1.54) | 0.030 | 95% | |
|  | (0-27 days) | Medium | 819 | 12.43 | 1.06 (0.87-1.30) | 0.538 | 0.97 (0.80-1.18) | 0.796 | 1.09 (0.90-1.33) | 0.385 | - | |
|  |  | High | 436 | 10.18 | 1.00(reference) |  |  |  |  |  |  | |
|  | Postneonatal | Low | 358 | 0.75 | 1.44 (1.11-1.88) | 0.006 | 1.21 (0.91-1.60) | 0.184 | 1.19 (0.92-1.55) | 0.186 | 53% | |
|  | (28-364 days) | Medium | 397 | 0.48 | 1.07 (0.83-1.38) | 0.596 | 0.97 (0.74-1.27) | 0.833 | 1.10 (0.86-1.42) | 0.452 | - | |
|  |  | High | 202 | 0.38 | 1.00(reference) |  |  |  |  |  | |  |

^a^ Deaths due to congenital malformations: ICD-8 codes 740-759 and ICD-10 codes Q00-Q99.

^b^ Pys, person-years; TE, total effect; CDE, controlled direct effect; PE, portion eliminated; MRR, mortality rate ratio; proportion eliminated: = (MRR_TE_ – MRR_CDE_)/(MRR_TE_-1); proportion eliminated is only presented if the MRRs of CDE and PE were in the same direction; PTB, preterm birth; SGA; small for gestational age.
