# Supplementary material for: Mediating roles of preterm birth and restricted fetal growth in the relationship between maternal education and infant mortality: A Danish population-based cohort study
Source: PLoS Med. 2019 Jun 14;16(6):e1002831. doi: 10.1371/journal.pmed.1002831 (PMC6568398; doi:10.1371/journal.pmed.1002831)
Supplement: S8 Table — (DOCX) [file pmed.1002831.s010.docx]

**S8 Table. The contribution of preterm birth and small for gestational age in explaining the association between maternal education and infant mortality due to external causes ^ab^**

| **Mediator** | **Period** | **Education** | **No. of death** | **Rate/10^2^ pys** | **MRR_TE_** | ***P* value** | **MRR_CDE_** | ***P* value** | **MRR_PE_** | ***P* value** | **Proportion eliminated** | |
| --- | --- | --- | --- | --- | --- | --- | --- | --- | --- | --- | --- | --- |
| PTB | Infant | Low | 79 | 0.15 | 2.76 (1.56-4.87) | 0.000 | 2.55 (1.46-4.46) | 0.001 | 1.08 (0.61-1.91) | 0.780 | 12% | |
|  | (< 1 year) | Medium | 71 | 0.08 | 1.87 (1.07-3.28) | 0.029 | 1.77 (1.02-3.07) | 0.043 | 1.06 (0.60-1.85) | 0.849 | 11% | |
|  |  | High | 28 | 0.05 | 1.00(reference) |  |  |  |  |  |  | |
|  | Neonatal | Low | 10 | 0.26 | 3.60 (1.04-12.45) | 0.043 | 3.03 (0.83-11.09) | 0.095 | 1.19 (0.34-4.12) | 0.785 | 22% | |
|  | (0-27 days) | Medium | 9 | 0.14 | 2.08 (0.63-6.84) | 0.226 | 1.58 (0.46-5.46) | 0.470 | 1.32 (0.40-4.33) | 0.648 | 47% | |
|  |  | High | 4 | 0.09 | 1.00(reference) |  |  |  |  |  |  | |
|  | Postneonatal | Low | 69 | 0.14 | 2.65 (1.42-4.94) | 0.002 | 2.48 (1.34-4.58) | 0.004 | 1.07 (0.57-1.99) | 0.834 | 10% | |
|  | (28-364 days) | Medium | 62 | 0.08 | 1.84 (0.99-3.41) | 0.053 | 1.80 (0.98-3.31) | 0.058 | 1.02 (0.55-1.89) | 0.946 | 5% | |
|  |  | High | 24 | 0.04 | 1.00(reference) |  |  |  |  |  |  | |
| SGA | Infant | Low | 79 | 0.15 | 2.76 (1.56-4.87) | 0.000 | 2.70 (1.52-4.78) | 0.001 | 1.02 (0.58-1.81) | 0.936 | 4% | |
|  | (< 1 year) | Medium | 71 | 0.08 | 1.87 (1.07-3.28) | 0.029 | 1.88 (1.07-3.32) | 0.029 | 0.99 (0.57-1.74) | 0.979 | - | |
|  |  | High | 28 | 0.05 | 1.00(reference) |  |  |  |  |  |  | |
|  | Neonatal | Low | 10 | 0.26 | 3.60 (1.04-12.45) | 0.043 | 3.30 (0.96-11.34) | 0.058 | 1.09 (0.31-3.78) | 0.891 | 11% | |
|  | (0-27 days) | Medium | 9 | 0.14 | 2.08 (0.63-6.84) | 0.226 | 2.02 (0.61-6.65) | 0.248 | 1.03 (0.31-3.39) | 0.959 | 6% | |
|  |  | High | 4 | 0.09 | 1.00(reference) |  |  |  |  |  |  | |
|  | Postneonatal | Low | 69 | 0.14 | 2.65 (1.42-4.94) | 0.002 | 2.62 (1.40-4.91) | 0.003 | 1.01 (0.54-1.88) | 0.971 | 2% | |
|  | (28-364 days) | Medium | 62 | 0.08 | 1.84 (0.99-3.41) | 0.053 | 1.86 (1.00-3.47) | 0.049 | 0.99 (0.53-1.83) | 0.968 | - | |
|  |  | High | 24 | 0.04 | 1.00(reference) |  |  |  |  |  |  | |
| PTB | Infant | Low | 79 | 0.15 | 2.76 (1.56-4.87) | 0.000 | 2.50 (1.42-4.39) | 0.001 | 1.11 (0.63-1.95) | 0.729 | 15% | |
| and | (< 1 year) | Medium | 71 | 0.08 | 1.87 (1.07-3.28) | 0.029 | 1.79 (1.03-3.13) | 0.040 | 1.04 (0.59-1.83) | 0.886 | 9% | |
| SGA |  | High | 28 | 0.05 | 1.00(reference) |  |  |  |  |  |  | |
|  | Neonatal | Low | 10 | 0.26 | 3.60 (1.04-12.45) | 0.043 | 2.71 (0.74-9.85) | 0.131 | 1.33 (0.38-4.60) | 0.654 | 34% | |
|  | (0-27 days) | Medium | 9 | 0.14 | 2.08 (0.63-6.84) | 0.226 | 1.60 (0.46-5.56) | 0.458 | 1.30 (0.40-4.27) | 0.665 | 44% | |
|  |  | High | 4 | 0.09 | 1.00(reference) |  |  |  |  |  |  | |
|  | Postneonatal | Low | 69 | 0.14 | 2.65 (1.42-4.94) | 0.002 | 2.47 (1.33-4.59) | 0.004 | 1.07 (0.58-2.00) | 0.826 | 11% | |
|  | (28-364 days) | Medium | 62 | 0.08 | 1.84 (0.99-3.41) | 0.053 | 1.83 (0.99-3.37) | 0.054 | 1.01 (0.54-1.87) | 0.979 | 2% | |
|  |  | High | 24 | 0.04 | 1.00(reference) |  |  |  |  |  | |  |

^a^ Deaths due to external causes: ICD-8 codes E800-E999, and ICD-10 codes V01-Y98.

^b^ Pys, person-years; TE, total effect; CDE, controlled direct effect; PE, portion eliminated; MRR, mortality rate ratio; proportion eliminated: = (MRR_TE_ – MRR_CDE_)/(MRR_TE_-1); proportion eliminated is only presented if the MRRs of CDE and PE were in the same direction; PTB, preterm birth; SGA; small for gestational age.
