# Supplementary material for: Mediating roles of preterm birth and restricted fetal growth in the relationship between maternal education and infant mortality: A Danish population-based cohort study
Source: PLoS Med. 2019 Jun 14;16(6):e1002831. doi: 10.1371/journal.pmed.1002831 (PMC6568398; doi:10.1371/journal.pmed.1002831)
Supplement: S9 Table — (DOCX) [file pmed.1002831.s011.docx]

**S9 Table. Sensitivity analyses of the influence of uncontrolled mediator-outcome confounding ^abc^**

| **Mediator** | **Period** | **Education** | **Original results** | | | **Mild unmeasured confounding (MRR_U_=1.5)** | | | **Strong unmeasured confounding (MRR_U_=2.5)** | | |
| --- | --- | --- | --- | --- | --- | --- | --- | --- | --- | --- | --- |
|  |  |  | **MRR_CDE_** | **MRR_PE_** | **Proportion eliminated** | **MRR_CDE_** | **MRR_PE_** | **Proportion eliminated** | **MRR_CDE_** | **MRR_PE_** | **Proportion eliminated** |
| PTB | Infant | Low | 1.34 | 1.22 | 46% | 1.46 | 1.11 | 27% | 1.65 | 0.99 | - |
|  | (< 1 year) | Medium | 1.10 | 1.08 | 48% | 1.15 | 1.04 | 23% | 1.21 | 0.98 | - |
|  |  | High | 1.00(ref.) |  |  |  |  |  |  |  |  |
|  | Neonatal | Low | 1.20 | 1.30 | 64% | 1.31 | 1.20 | 45% | 1.48 | 1.06 | 16% |
|  | (0-27 days) | Medium | 1.07 | 1.11 | 64% | 1.11 | 1.06 | 38% | 1.18 | 1.00 | 3% |
|  |  | High | 1.00(ref.) |  |  |  |  |  |  |  |  |
|  | Postneonatal | Low | 1.62 | 1.08 | 17% | 1.76 | 0.99 | - | 1.99 | 0.88 | - |
|  | (28-364 days) | Medium | 1.17 | 1.04 | 20% | 1.22 | 0.99 | - | 1.29 | 0.94 | - |
|  |  | High | 1.00(ref.) |  |  |  |  |  |  |  |  |
| SGA | Infant | Low | 1.56 | 1.05 | 11% | 1.70 | 0.96 | - | 1.92 | 0.85 | - |
|  | (< 1 year) | Medium | 1.17 | 1.02 | 13% | 1.22 | 0.98 | - | 1.29 | 0.93 | - |
|  |  | High | 1.00(ref.) |  |  |  |  |  |  |  |  |
|  | Neonatal | Low | 1.50 | 1.05 | 12% | 1.63 | 0.96 | - | 1.84 | 0.85 | - |
|  | (0-27 days) | Medium | 1.15 | 1.03 | 17% | 1.20 | 0.98 | - | 1.27 | 0.93 | - |
|  |  | High | 1.00(ref.) |  |  |  |  |  |  |  |  |
|  | Postneonatal | Low | 1.68 | 1.04 | 8% | 1.84 | 0.95 | - | 2.07 | 0.84 | - |
|  | (28-364 days) | Medium | 1.20 | 1.01 | 6% | 1.25 | 0.97 | - | 1.32 | 0.92 | - |
|  |  | High | 1.00(ref.) |  |  |  |  |  |  |  |  |
| PTB | Infant | Low | 1.28 | 1.28 | 55% | 1.39 | 1.17 | 38% | 1.57 | 1.04 | 9% |
| and | (< 1 year) | Medium | 1.08 | 1.10 | 60% | 1.13 | 1.06 | 35% | 1.19 | 1.00 | - |
| SGA |  | High | 1.00(ref.) |  |  |  |  |  |  |  |  |
|  | Neonatal | Low | 1.14 | 1.38 | 75% | 1.24 | 1.26 | 58% | 1.40 | 1.12 | 29% |
|  | (0-27 days) | Medium | 1.04 | 1.14 | 81% | 1.08 | 1.09 | 54% | 1.15 | 1.03 | 19% |
|  |  | High | 1.00(ref.) |  |  |  |  |  |  |  |  |
|  | Postneonatal | Low | 1.57 | 1.11 | 23% | 1.71 | 1.02 | 5% | 1.93 | 0.91 | - |
|  | (28-364 days) | Medium | 1.16 | 1.04 | 21% | 1.21 | 1.00 | - | 1.28 | 0.94 | - |
|  |  | High | 1.00(ref.) |  |  |  |  |  |  |  |  |

^a^ CDE, controlled direct effect; PE, portion eliminated; MRR, mortality rate ratio; proportion eliminated: = (MRR_TE_ – MRR_CDE_)/(MRR_TE_-1); proportion eliminated is only presented if the MRRs of CDE and PE were in the same direction; PTB, preterm birth; SGA; small for gestational age.

^b^ Original results: the primary result of this study (Table 2).

^c^ MRR*_U_*: denotes the effect if unmeasured confounding U increased the likelihood of the mortality by a factor of 1.5 or 2.5.
