# Supplementary material for: Mediating roles of preterm birth and restricted fetal growth in the relationship between maternal education and infant mortality: A Danish population-based cohort study
Source: PLoS Med. 2019 Jun 14;16(6):e1002831. doi: 10.1371/journal.pmed.1002831 (PMC6568398; doi:10.1371/journal.pmed.1002831)
Supplement: S10 Table — (DOCX) [file pmed.1002831.s012.docx]

**S10 Table. The contribution of preterm birth and small for gestational age in explaining the association between maternal education and infant mortality with an additional adjustment for maternal country of origin ^a^**

| **Mediator** | **Period** | **Education** | **MRR_TE_** | ***P* value** | **MRR_CDE_** | ***P* value** | **MRR_PE_** | ***P* value** | | **Proportion eliminated** |
| --- | --- | --- | --- | --- | --- | --- | --- | --- | --- | --- |
| PTB | Infant | Low | 1.63 (1.48-1.80) | 0.000 | 1.36 (1.18-1.55) | 0.000 | 1.20 (1.06-1.36) | 0.003 | | 44% |
|  | (< 1 year) | Medium | 1.19 (1.08-1.31) | 0.000 | 1.11 (0.97-1.27) | 0.125 | 1.07 (0.95-1.21) | 0.261 | | 42% |
|  |  | High | 1.00(reference) |  |  |  |  |  | |  |
|  | Neonatal | Low | 1.57 (1.38-1.78) | 0.000 | 1.21 (1.01-1.45) | 0.040 | 1.29 (1.09-1.52) | 0.002 | | 63% |
|  | (0-27 days) | Medium | 1.18 (1.05-1.33) | 0.006 | 1.07 (0.90-1.27) | 0.447 | 1.09 (0.94-1.28) | 0.260 | | 59% |
|  |  | High | 1.00(reference) |  |  |  |  |  | |  |
|  | Postneonatal | Low | 1.75 (1.49-2.04) | 0.000 | 1.58 (1.30-1.92) | 0.000 | 1.12 (0.94-1.34) | 0.207 | | 25% |
|  | (28-364 days) | Medium | 1.21 (1.04-1.41) | 0.015 | 1.15 (0.95-1.40) | 0.148 | 1.06 (0.89-1.26) | 0.524 | | 31% |
|  |  | High | 1.00(reference) |  |  |  |  |  | |  |
| SGA | Infant | Low | 1.63 (1.48-1.80) | 0.000 | 1.57 (1.39-1.77) | 0.000 | 1.04 (0.92-1.18) | 0.533 | | 10% |
|  | (< 1 year) | Medium | 1.19 (1.08-1.31) | 0.000 | 1.16 (1.03-1.31) | 0.011 | 1.02 (0.91-1.15) | 0.733 | | 13% |
|  |  | High | 1.00(reference) |  |  |  |  |  | |  |
|  | Neonatal | Low | 1.57 (1.38-1.78) | 0.000 | 1.49 (1.27-1.76) | 0.000 | 1.04 (0.89-1.23) | 0.620 | | 11% |
|  | (0-27 days) | Medium | 1.18 (1.05-1.33) | 0.006 | 1.14 (0.98-1.33) | 0.101 | 1.03 (0.88-1.20) | 0.726 | | 19% |
|  |  | High | 1.00(reference) |  |  |  |  |  | |  |
|  | Postneonatal | Low | 1.75 (1.49-2.04) | 0.000 | 1.72 (1.44-2.05) | 0.000 | 1.03 (0.86-1.23) | 0.745 | | 7% |
|  | (28-364 days) | Medium | 1.21 (1.04-1.41) | 0.015 | 1.21 (1.02-1.44) | 0.028 | 1.01 (0.84-1.20) | 0.947 | | 3% |
|  |  | High | 1.00(reference) |  |  |  |  |  | |  |
| PTB | Infant | Low | 1.63 (1.48-1.80) | 0.000 | 1.29 (1.13-1.47) | 0.000 | 1.26 (1.12-1.43) | 0.000 | | 54% |
| and | (< 1 year) | Medium | 1.19 (1.08-1.31) | 0.000 | 1.08 (0.95-1.23) | 0.217 | 1.10 (0.97-1.23) | 0.132 | | 55% |
| SGA |  | High | 1.00(reference) |  |  |  |  |  | |  |
|  | Neonatal | Low | 1.57 (1.38-1.78) | 0.000 | 1.14 (0.96-1.36) | 0.139 | 1.36 (1.16-1.61) | 0.000 | | 75% |
|  | (0-27 days) | Medium | 1.18 (1.05-1.33) | 0.006 | 1.04 (0.88-1.23) | 0.680 | 1.13 (0.97-1.32) | 0.125 | | 79% |
|  |  | High | 1.00(reference) |  |  |  |  |  | |  |
|  | Postneonatal | Low | 1.75 (1.49-2.04) | 0.000 | 1.61 (1.33-1.93) | 0.000 | 1.10 (0.92-1.31) | 0.285 | | 21% |
|  | (28-364 days) | Medium | 1.21 (1.04-1.41) | 0.015 | 1.19 (0.99-1.42) | 0.067 | 1.03 (0.86-1.23) | 0.741 | | 16% |
|  |  | High | 1.00(reference) |  |  |  |  | |  |  |

^a^ Pys, person-years; TE, total effect; CDE, controlled direct effect; PE, portion eliminated; MRR, mortality rate ratio; proportion eliminated: = (MRR_TE_ – MRR_CDE_)/(MRR_TE_-1); proportion eliminated is only presented if the MRRs of CDE and PE were in the same direction; PTB, preterm birth; SGA; small for gestational age.
