# Supplementary material for: Mediating roles of preterm birth and restricted fetal growth in the relationship between maternal education and infant mortality: A Danish population-based cohort study
Source: PLoS Med. 2019 Jun 14;16(6):e1002831. doi: 10.1371/journal.pmed.1002831 (PMC6568398; doi:10.1371/journal.pmed.1002831)
Supplement: S11 Table — (DOCX) [file pmed.1002831.s013.docx]

**S11 Table. The contribution of preterm birth and small for gestational age in explaining the association between maternal education and infant mortality using the traditional approach with and without mediators for mediation analysis ^a^**

| **Mediator** | **Period** | **Education** | **MRR_TE_** | ***P* value** | **MRR_CDE_** | ***P* value** | **MRR_PE_** | ***P* value** | | **Proportion eliminated** |
| --- | --- | --- | --- | --- | --- | --- | --- | --- | --- | --- |
| PTB | Infant | Low | 1.63 (1.48-1.80) | 0.000 | 1.17 (1.09-1.25) | 0.000 | 1.40 (1.27-1.54) | 0.000 | | 74% |
|  | (< 1 year) | Medium | 1.19 (1.08-1.31) | 0.000 | 1.01 (0.96-1.08) | 0.636 | 1.17 (1.07-1.29) | 0.001 | | 92% |
|  |  | High | 1.00(reference) |  |  |  |  |  | |  |
|  | Neonatal | Low | 1.57 (1.38-1.78) | 0.000 | 1.09 (1.00-1.18) | 0.049 | 1.44 (1.27-1.63) | 0.000 | | 85% |
|  | (0-27 days) | Medium | 1.18 (1.05-1.33) | 0.006 | 1.02 (0.95-1.10) | 0.632 | 1.16 (1.03-1.31) | 0.014 | | 90% |
|  |  | High | 1.00(reference) |  |  |  |  |  | |  |
|  | Postneonatal | Low | 1.75 (1.49-2.04) | 0.000 | 1.31 (1.17-1.47) | 0.000 | 1.33 (1.14-1.56) | 0.000 | | 58% |
|  | (28-364 days) | Medium | 1.21 (1.04-1.41) | 0.015 | 1.03 (0.92-1.14) | 0.632 | 1.18 (1.01-1.37) | 0.035 | | 88% |
|  |  | High | 1.00(reference) |  |  |  |  |  | |  |
| SGA | Infant | Low | 1.63 (1.48-1.80) | 0.000 | 1.32 (1.24-1.41) | 0.000 | 1.23 (1.12-1.36) | 0.000 | | 49% |
|  | (< 1 year) | Medium | 1.19 (1.08-1.31) | 0.000 | 1.08 (1.02-1.15) | 0.008 | 1.10 (1.00-1.21) | 0.051 | | 56% |
|  |  | High | 1.00(reference) |  |  |  |  |  | |  |
|  | Neonatal | Low | 1.57 (1.38-1.78) | 0.000 | 1.32 (1.21-1.43) | 0.000 | 1.19 (1.05-1.35) | 0.007 | | 44% |
|  | (0-27 days) | Medium | 1.18 (1.05-1.33) | 0.006 | 1.12 (1.04-1.20) | 0.004 | 1.06 (0.94-1.19) | 0.344 | | 36% |
|  |  | High | 1.00(reference) |  |  |  |  |  | |  |
|  | Postneonatal | Low | 1.75 (1.49-2.04) | 0.000 | 1.32 (1.18-1.48) | 0.000 | 1.32 (1.13-1.54) | 0.000 | | 57% |
|  | (28-364 days) | Medium | 1.21 (1.04-1.41) | 0.015 | 1.04 (0.93-1.15) | 0.512 | 1.17 (1.00-1.36) | 0.048 | | 83% |
|  |  | High | 1.00(reference) |  |  |  |  |  | |  |
| PTB | Infant | Low | 1.63 (1.48-1.80) | 0.000 | 1.10 (1.02-1.18) | 0.011 | 1.49 (1.35-1.64) | 0.000 | | 85% |
| and | (< 1 year) | Medium | 1.19 (1.08-1.31) | 0.000 | 0.99 (0.93-1.06) | 0.847 | 1.20 (1.09-1.32) | 0.000 | | - |
| SGA |  | High | 1.00(reference) |  |  |  |  |  | |  |
|  | Neonatal | Low | 1.57 (1.38-1.78) | 0.000 | 1.00 (0.91-1.09) | 0.993 | 1.57 (1.38-1.78) | 0.000 | | - |
|  | (0-27 days) | Medium | 1.18 (1.05-1.33) | 0.006 | 0.99 (0.92-1.07) | 0.869 | 1.19 (1.06-1.34) | 0.004 | | - |
|  |  | High | 1.00(reference) |  |  |  |  |  | |  |
|  | Postneonatal | Low | 1.75 (1.49-2.04) | 0.000 | 1.27 (1.13-1.42) | 0.000 | 1.38 (1.18-1.61) | 0.000 | | 64% |
|  | (28-364 days) | Medium | 1.21 (1.04-1.41) | 0.015 | 1.01 (0.91-1.13) | 0.796 | 1.19 (1.02-1.39) | 0.024 | | 93% |
|  |  | High | 1.00(reference) |  |  |  |  | |  |  |

^a^ Pys, person-years; TE, total effect; CDE, controlled direct effect; PE, portion eliminated; MRR, mortality rate ratio; proportion eliminated: = (MRR_TE_ – MRR_CDE_)/(MRR_TE_-1); proportion eliminated is only presented if the MRRs of CDE and PE were in the same direction; PTB, preterm birth; SGA; small for gestational age.
