# Supplementary material for: Mediating roles of preterm birth and restricted fetal growth in the relationship between maternal education and infant mortality: A Danish population-based cohort study
Source: PLoS Med. 2019 Jun 14;16(6):e1002831. doi: 10.1371/journal.pmed.1002831 (PMC6568398; doi:10.1371/journal.pmed.1002831)
Supplement: S1 Text — (DOCX) [file pmed.1002831.s014.docx]

**S1 Text.**

**Data analysis plan for:**

Mediating roles of preterm birth and restricted fetal growth in the relationship between maternal education and infant mortality: a Danish population-based cohort study

# Background

Infant mortality in high-income countries has decreased during the last decades.[[1](#_ENREF_1)] Nevertheless, the observed socioeconomic disparities in infant mortality persist in high-income countries [[2-7](#_ENREF_2)] and may even be increasing in specific populations.[[8](#_ENREF_8), [9](#_ENREF_9)] New understandings of the mechanisms that underline socioeconomic differences in infant deaths are essential to drive health initiatives to reduce potentially preventable deaths.

The previous studies have shown that prenatal factors, including preterm birth, low birth weight, and small for gestational age are associated not only with socioeconomic disadvantage[[6](#_ENREF_6), [10-14](#_ENREF_10)], but also with offspring mortality [[15-19](#_ENREF_15)]. However, there is a lack of research addressing the mediatory roles of these prenatal factors in the association between socioeconomic disadvantage and infant mortality using causal mediation analysis methods. In this study we aimed to use a causal mediation approach to quantify how much of socioeconomic disadvantage in infant mortality was mediated by preterm birth and small for gestational age in Denmark.

# Data sources

In Denmark, all live-born and new residents are assigned a unique civil personal identity code, which allows accurate individual information linkage from various national registries [[20](#_ENREF_20)].

# Study population and period

In Denmark, all singletons born between 1^st^ Jan 1981 to 31^st^ December 2015 will be included. Children will be followed from the birth until death, their 1^st^ birthday, emigration or the end of the study period on (December 31^st^ 2016), whichever occurs first.

# Variable definitions

## Outcomes

- All-cause infant mortality (0-364 days)
  - Neonatal mortality (0-27 days), and postneonatal mortality (28-364 days).
- Cause-specific mortality
  - Death due to diseases and medical conditions [ICD-8 codes 000-799, and ICD-10 codes A00-R99], or death due to external causes [ICD-8 codes E800-E999, and ICD-10 codes V01-Y98]
  - death due to certain conditions originating in the perinatal period [ICD-8 codes 760-779 and ICD-10 P00-P96], or death due to congenital malformations [ICD-8 740-759 and ICD-10 Q00-Q99]

## Exposure

- Maternal education was measured with the highest level of education attained at childbirth and categorized as low (primary and lower secondary education), medium (upper secondary education and academy profession degree) or high (university education at bachelor’s degree level or higher).

## Mediators

- A dichotomous mediator in the main analysis: preterm birth (PTB: yes, no) and small for gestational age (SGA: yes, no)
- We also used finer categorizations of PTB (<28, 28-31, 32-36, 37+ weeks) and SGA (birthweight below the 3^rd^, between 3^rd^ and 10^th^, above the 10^th^ percentile for infants of the same gestational age, sex and birth year).

## Covariates

- Sex
- Year of birth
- Maternal age
- Maternal smoking during pregnancy: coded into a binary variable 1= yes, 0=no; available period: 1991-2016
- Major congenital anomalies: Yes, No
- Maternal cohabitation: yes, no
- Maternal residence: yes, no

As the information on some variables was only available in some specific time periods, we created missing indicators for the variables with missing values.

# Statistical analyses

The approach for causal mediation analysis was based on a counterfactual framework whereby the total effect (TE) can be decomposed into controlled direct effect (CDE) and portion eliminated (PE).[[21](#_ENREF_21), [22](#_ENREF_22)] TE and CDE were estimated using inverse-probability-weighted marginal structural models (MSMs).[[23](#_ENREF_23)] The proportion of the total effect eliminated through the two mediators or the proportion eliminated[[24](#_ENREF_24)] was reported if the directions of CDE and PE were the same.[[25](#_ENREF_25)]

We first assessed the mediating role of PTB and SGA separately and then analyzed PTB and SGA together as a joint mediator. We also performed mediation analysis according to birth year of the offspring.

We performed sensitivity analysis to assess and adjust for violations in the uncontrolled confounding assumptions. [[26](#_ENREF_26)]

*We examined the mediating roles of PTB in non-SGA infants and the mediating role of SGA in term-born infants (added during revision).*

*We also performed mediation analysis using traditional approach[*[*27*](#_ENREF_27)*] (added during revision).*

# References

1. Viner, R.M., et al., *Deaths in young people aged 0-24 years in the UK compared with the EU15+ countries, 1970-2008: analysis of the WHO Mortality Database.* Lancet, 2014. **384**(9946): p. 880-892.

2. Calling, S., et al., *Socioeconomic inequalities and infant mortality of 46,470 preterm infants born in Sweden between 1992 and 2006.* Paediatric and Perinatal Epidemiology, 2011. **25**(4): p. 357-365.

3. Petrou, S., et al., *Social class inequalities in childhood mortality and morbidity in an English population.* Paediatric and Perinatal Epidemiology, 2006. **20**(1): p. 14-23.

4. Cammu, H., et al., *The higher the educational level of the first-time mother, the lower the fetal and post-neonatal but not the neonatal mortality in Belgium (Flanders).* European Journal of Obstetrics, Gynecology, and Reproductive Biology, 2010. **148**(1): p. 13-16.

5. Arntzen, A., et al., *Post-neonatal mortality in Norway 1969-95: a cause-specific analysis.* International Journal of Epidemiology, 2006. **35**(4): p. 1083-1089.

6. Wood, A.M., et al., *Trends in socioeconomic inequalities in risk of sudden infant death syndrome, other causes of infant mortality, and stillbirth in Scotland: population based study.* BMJ, 2012. **344**: p. e1552.

7. Arntzen, A., et al., *Socioeconomic status and risk of infant death. A population-based study of trends in Norway, 1967-1998.* International Journal of Epidemiology, 2004. **33**(2): p. 279-288.

8. Strand, B.H., et al., *Educational inequalities in mortality over four decades in Norway: prospective study of middle aged men and women followed for cause specific mortality, 1960-2000.* BMJ, 2010. **340**: p. c654.

9. Mackenbach, J.P., et al., *Widening socioeconomic inequalities in mortality in six Western European countries.* International Journal of Epidemiology, 2003. **32**(5): p. 830-837.

10. Weightman, A.L., et al., *Social inequality and infant health in the UK: systematic review and meta-analyses.* BMJ Open, 2012. **2**(3).

11. Odd, D., et al., *Risk of low Apgar scores and socioeconomic status over a 30-year period.* Journal of Maternal-Fetal & Neonatal Medicine, 2014. **27**(6): p. 603-607.

12. Odd, D.E., et al., *Risk of low Apgar score and socioeconomic position: a study of Swedish male births.* Acta Paediatrica, 2008. **97**(9): p. 1275-1280.

13. Ekert-Jaffe, O., et al., *Fecondite, calendrier des naissances et milieu social en France et en Grande-Bretagne: Politiques sociales et polarisation socioprofessionnelle.* Population (French Edition), 2002. **57**(3): p. 485-518.

14. Rindfuss, R.R. and C. St. John, *Social determinants of age at first birth.* Journal of Marriage and the Family, 1983: p. 553-565.

15. D'Onofrio, B.M., et al., *Preterm birth and mortality and morbidity: a population-based quasi-experimental study.* JAMA Psychiatry, 2013. **70**(11): p. 1231-1240.

16. McCormick, M.C., *The contribution of low birth weight to infant mortality and childhood morbidity.* New England Journal of Medicine, 1985. **312**(2): p. 82-90.

17. Iliodromiti, S., et al., *Apgar score and the risk of cause-specific infant mortality: a population-based cohort study.* Lancet, 2014. **384**(9956): p. 1749-1755.

18. Hart, C.L., et al., *Childhood IQ, social class, deprivation, and their relationships with mortality and morbidity risk in later life: Prospective observational study linking the Scottish Mental Survey 1932 and the Midspan studies.* Psychosomatic Medicine, 2003. **65**(5): p. 877-883.

19. Jacobsson, B., L. Ladfors, and I. Milsom, *Advanced maternal age and adverse perinatal outcome.* Obstetrics and Gynecology, 2004. **104**(4): p. 727-733.

20. Li, J., et al., *Cohort profile: the Nordic Perinatal Bereavement Cohort.* International Journal of Epidemiology, 2011. **40**(5): p. 1161-1167.

21. Robins, J.M. and S. Greenland, *Identifiability and exchangeability for direct and indirect effects.* Epidemiology, 1992. **3**(2): p. 143-155.

22. Pearl, J. *Direct and indirect effects*. in *Proceedings of the seventeenth conference on uncertainty in artificial intelligence*. 2001. Morgan Kaufmann Publishers Inc.

23. Robins, J.M., M.A. Hernan, and B. Brumback, *Marginal structural models and causal inference in epidemiology.* Epidemiology, 2000. **11**(5): p. 550-560.

24. VanderWeele, T.J., *Policy-relevant proportions for direct effects.* Epidemiology, 2013. **24**(1): p. 175-176.

25. Vanderweele, T.J. and S. Vansteelandt, *Odds ratios for mediation analysis for a dichotomous outcome.* American Journal of Epidemiology, 2010. **172**(12): p. 1339-1348.

26. VanderWeele, T.J., *Bias formulas for sensitivity analysis for direct and indirect effects.* Epidemiology, 2010. **21**(4): p. 540-551.

27. Baron, R.M. and D.A. Kenny, *The moderator–mediator variable distinction in social psychological research: Conceptual, strategic, and statistical considerations.* Journal of Personality and Social Psychology, 1986. **51**(6): p. 1173.
