# Supplementary material for: Mediating roles of preterm birth and restricted fetal growth in the relationship between maternal education and infant mortality: A Danish population-based cohort study
Source: PLoS Med. 2019 Jun 14;16(6):e1002831. doi: 10.1371/journal.pmed.1002831 (PMC6568398; doi:10.1371/journal.pmed.1002831)
Supplement: S2 Text — (DOCX) [file pmed.1002831.s015.docx]

**S2 Text. A detailed description of the inverse probability weighting approach for causal mediation analysis**

The approach for causal mediation analysis was based on a counterfactual framework whereby the total effect (TE) can be decomposed into controlled direct effect (CDE) and portion eliminated (PE) [1–3]. The CDE captured the influence of maternal education on infant (neonatal and postneonatal) mortality if the link between maternal education and the mediator (PTB or SGA) was prevented or removed hypothetically. This simulated a scenario wherein the sample distributions of the mediator were no longer dependent on maternal education. PE, the difference between TE and CDE, measured the portion of the TE of maternal education that would be eliminated by eliminating the mediator. TE and CDE were estimated using inverse-probability-weighted marginal structural models (MSMs) [4]. For the MSM for the TE, we used weighted regressions of infant mortality on maternal education. The weight, which is called the inverse-probability-of-treatment weight (IPTW), was estimated for each mother in the sample as the ratio of (i) the estimated marginal probability of the mother’s actual educational attainment to (ii) the estimated probability of each mother’s actual educational attainment conditional on their aforementioned covariates (excluding maternal smoking, offspring sex, congenital malformation of offspring, PTB, and SGA). The IPTW simulates the scenario wherein these covariates, which could be confounders, are no longer associated with maternal education, thus eliminating any confounding by these covariates. To estimate the CDE, the corresponding MSM used a product of the IPTW for maternal education and an additional inverse-probability-of-mediators weight (IPMW). The IPMW was estimated for each infant in the sample as the ratio of (i) the estimated marginal probability of the infant’s actual PTB and SGA to (ii) the estimated probability of each infant’s actual PTB and SGA conditional on their aforementioned covariates. The IPMW simulates the scenario wherein the mediators (PTB and SGA) are no longer associated with maternal education, thus eliminating any mediation by the mediators. The PE was subsequently estimated from the model for TE offsetting the estimated CDE. We considered possible exposure–mediator and mediator–mediator interactions. As the mediator–mediator interactions in causal mediation analysis with multiple mediators were null, the final models included only exposure–mediator interactions. We estimated mortality rate ratios (MRRs) with their 95% confidence intervals (CIs) based on robust variance estimation. The proportion of the TE eliminated through the 2 mediators, i.e., the proportion eliminated ([MRR_TE_ – MRR_CDE_]/[MRR_TE_ ­– 1]), was reported if the MRRs of CDE and PE were in the same direction [1,5]. We first assessed the mediating role of PTB and SGA separately, i.e., one mediator at a time. Then we analyzed PTB and SGA together as a joint mediator, i.e., not separating their individual contributions.

We used causal diagram below to depict how to use inverse-probability-weighted marginal structure model approach to estimate TE, CDE and PE of maternal education on education.

Figure. Directed acyclic graph for the structural causal mediation model of the relationship between the exposure A and the outcome Y

Let *A, M (M1, M2)* and *Y* denote exposure, mediators and outcome, respectively. Let *C1* denote a vector of exposure-outcome confounders, and mediator-outcome confounders which are not affected by the exposure. Let *C2* denote the vector of mediator-outcome confounders affected by the exposure.

**Step 1:** Fit a logistic regression model for exposure and compute inverse-probability-of-treatment weight (IPTW) for total effect.

$$IPTW=\frac{P(A)}{P(A|C1)}$$

Then, fit IPTW weighted marginal structural models to estimate total effect (TE) of exposure on outcome.

$$E\left( Y \right)=exp(\alpha_{0}+\alpha_{a}\cdot A)$$

$exp(\alpha_{a})$ represents the TE of exposure on outcome.

**Step 2:** Fit logistic regression models for mediators to compute inverse-probability-of-mediator weight (IPMW) and then multiplied by IPTW to compute weight for controlled direct effect (CDE) of exposure on outcome not through mediators M1 and M2.

$$IPMW=\frac{P(M_{1})}{P(M_{1}|A,C1, C2)}\cdot\frac{P(M_{2}|M_{1})}{P(M_{2}|M_{1},A,C1,C2)}=\frac{P(M_{2},M_{1})}{P(M_{2}, M_{1}|A,C1,C2)}$$

$$Weight for CDE=IPTW\cdot IPMW=\frac{P(A)}{P(A|C1)}\cdot\frac{P(M_{2},M_{1})}{P(M_{2}, M_{1}|A,C1,C2)}$$

Then, fit IPTW·IPMW weighted marginal structural models to estimate CDE of exposure on outcome, which allows the possible exposure-mediator and mediator-mediator interactions.

$$E\left( Y \right)={exp(\beta}_{0}+\beta_{a}\cdot A+\beta_{m1}\cdot M_{1}+\beta_{m2}\cdot M_{2}+\beta_{am1}\cdot A\cdot M_{1}+\beta_{am2}\cdot A\cdot M_{2}+\beta_{m1m2}\cdot M_{1}\cdot M_{2}+\beta_{am1m2}\cdot A\cdot{M_{1}\cdot M}_{2})$$

If exposure-mediator and mediator-mediator interactions are towards the null,the CDE can be estimated by $E\left( Y \right)={exp(\beta}_{0}+\beta_{a}\cdot A+\beta_{m1}\cdot M_{1}+\beta_{m2}\cdot M_{2})$,

${exp(\beta}_{a})$ represents the CDE of exposure on outcome.

In our illustrated DAG, we assume M1 temporally succeeds M2. However, when M2 temporally precedes M1, we still get the same formula for the weight for CDE. It indicates CDE does not depend on the ordering of the mediators when we estimated the combined mediating effect of M1 and M2 on the association between exposure and outcome using weighting approach presented in our study.

If there is one mediator, the weight IPMW is simplified to $\frac{P(M_{1})}{P(M_{1}|A,C1, C2)}$

**Step3:** The portion eliminated (PE) was subsequently estimated from the model for TE offsetting the estimated CDE.

**Reference**

1. VanderWeele T. Explanation in causal inference: methods for mediation and interaction: Oxford University Press; 2015.
2. Pearl J, editor Direct and indirect effects. Proceedings of the seventeenth conference on uncertainty in artificial intelligence; 2001: Morgan Kaufmann Publishers Inc.
3. Robins JM, Greenland S. Identifiability and exchangeability for direct and indirect effects. Epidemiology. 1992;3(2):143-55. Epub 1992/03/01. PubMed PMID: 1576220.
4. Robins JM, Hernan MA, Brumback B. Marginal structural models and causal inference in epidemiology. Epidemiology. 2000;11(5):550-60. Epub 2000/08/24. PubMed PMID: 10955408.
5. VanderWeele TJ. Policy-relevant proportions for direct effects. Epidemiology. 2013;24(1):175-6. Epub 2012/12/13. doi: 10.1097/EDE.0b013e3182781410. PubMed PMID: 23232624; PubMed Central PMCID: PMCPMC3523303.
